# Epigenetic regulation-mediated disorders in dopamine transporter endocytosis: A novel mechanism for the pathogenesis of Parkinson's disease

**DOI:** 10.7150/thno.107436

**Published:** 2025-01-13

**Authors:** Ziqi Liang, Wanqing Liu, Mian Cao, Jiajun Cui, Jinshuai Lan, Yue Ding, Tong Zhang, Zizhao Yang

**Affiliations:** 1 School of Pharmacy, Shanghai University of Traditional Chinese Medicine, Shanghai, 201210, China.; 2 Program in Neuroscience and Behavioral Disorders, Duke-NUS Medical School, Singapore, 169857, Singapore; Department of Physiology, National University of Singapore, Singapore, 169857, Singapore.; 3 Department of Biochemistry, College of Medicine, Yichun University, Yichun, Jiangxi 336000, China.; 4 School of Pharmacy, Shanghai University of Traditional Chinese Medicine, Shanghai, 201210, China.; 5 Department of General Surgery, Seventh People's Hospital of Shanghai University of Traditional Chinese Medicine, Shanghai, 200137, China.

**Keywords:** Parkinson's Disease (PD), Dopamine Transporter (DAT) Endocytosis, Epigenetic Regulations, Protein Kinase C (PKC) Pathway, Brain-derived Neurotrophic Factor (BDNF)

## Abstract

Mechanisms such as DNA methylation, histone modifications, and non-coding RNA regulation may impact the endocytosis of dopamine transporter (DAT) by influencing processes like neuronal survival, thereby contributing to the initiation and progression of Parkinson's Disease (PD). Some small molecule inhibitors or natural bioactive compounds have the potential to modulate epigenetic processes, thereby reversing induced pluripotent stem cells (iPSCs) reprogramming and abnormal differentiation, offering potential therapeutic effects for PD. Although no specific DNA modification enzyme directly regulates DAT endocytosis, enzymes such as DNA methyltransferases (DNMTs) may indirectly influence DAT endocytosis by regulating the expression of genes associated with this process. DNA modifications impact DAT endocytosis by modulating key signaling pathways, including the (protein kinase C) PKC and D2 receptor (D2R) pathways. Key enzymes involved in RNA modifications that influence DAT endocytosis include m^6^A methyltransferases and other related enzymes. This regulation impacts the synthesis and function of proteins involved in DAT endocytosis, thereby indirectly affecting the process itself. RNA modifications regulate DAT endocytosis through various indirect pathways, as well as histone modifications. Key enzymes influence the expression of genes associated with DAT endocytosis by modulating the chromatin's accessibility and compaction state. These enzymes control the expression of proteins involved in regulating endocytosis, promoting endosome formation, and facilitating recycling processes. Through the modulation exerted by these enzymes, the speed of DAT endocytosis and recycling patterns are indirectly regulated, establishing a crucial epigenetic control point for the regulation of neurotransmitter transport. Based on this understanding, we anticipate that targeting these processes could lead to favorable therapeutic effects for early PD pathogenesis.

## Introduction

The prevalence of Parkinson's disease (PD) ranks second among neurodegenerative disorders globally, following Alzheimer's disease (AD). It is estimated that the global PD population reaches approximately 10 million individuals, with its incidence strongly correlated to advancing age 1. The prevalence of this condition is typically higher among individuals aged 60 and above, with an estimated incidence rate of approximately 1 to 2 cases per 1,000 people in this age cohort 2. The condition is characterized by the progressive degeneration of dopaminergic (DAergic) neurons in the substantia nigra pars compacta, making it a neurodegenerative disorder 3. The aforementioned statement refers to a fundamental pathological alteration closely linked to the disease 4. The substantia nigra, a critical region in the brain responsible for motor control, primarily utilizes dopamine (DA) as its neurotransmitter. Motor symptoms of PD typically manifest only when there is a significant loss of DAergic neurons, usually exceeding 50-70% 5. In the brains of patients with PD, Lewy bodies 6, which are one of the pathological hallmarks, consisting of intracellular inclusions formed by the abnormal aggregation of α-synuclein (SNCA) 7. The precise etiology of PD remains incompletely elucidated; however, research indicates that neuroinflammation and oxidative stress may exert pivotal roles in the disease's progression. Excessive generation of free radicals and heightened immune responses can expedite neuronal damage and demise, thereby contributing to the degeneration of DAergic neurons in PD 8. Existing research suggests that the development of PD may be influenced by a combination of genetic factors, environmental factors, and neurobiological mechanisms. It has been observed that around 10-15% of individuals with PD have a family history, and several genes including SNCA, LRRK2, and PARK2 have been associated with this condition 9, 10. Mutations that enhance the kinase activity of LRRK2 constitute one of the most prevalent hereditary factors associated with PD 11, 12. Additionally, the onset of PD is also associated with certain environmental toxins such as pesticides and heavy metals. These substances have been found to induce oxidative stress and neurotoxicity, ultimately resulting in damage to DAergic neurons 13. Therefore, individuals residing in industrialized regions or those with prolonged exposure to specific chemicals may face an elevated susceptibility to the development of this disease. In addition to genetic and environmental factors, neurobiological mechanisms such as mitochondrial dysfunction, protein misfolding, and lysosomal dysfunction may also contribute to such pathogenesis 14.

The abnormal differentiation of DAergic neurons and the endocytosis of dopamine transporter (DAT) are closely associated with the onset and PD progression during the process of DA reduction in the nigrostriatal pathway 15. Under normal circumstances, DAT facilitates the reabsorption of DA from the synaptic cleft into the presynaptic cell through endocytosis, followed by either recycling or degradation. The regulation of movement within this pathway is attributed to the role of DA, and any abnormalities in DA synthesis, release, or signaling will lead to the characteristic motor symptoms, including tremor, rigidity, bradykinesia, and postural instability 16. Under normal circumstances, DAergic neurons in the substantia nigra release DA through synapses, which subsequently binds to receptors in the striatum to exert its physiological effects 17. The degeneration and death of neurons can be attributed to a reduction in DA synthesis and impaired release into the synapse, leading to decrease DA levels in the striatum and subsequent dysfunction in motor regulation 18. This aberrant differentiation can lead to early neuronal dysfunction and even expedite their demise, thereby facilitating the onset of PD 19. Jiang *et al.* have demonstrated that the inhibition of DAT in conjunction with elevated levels of Parkin leads to a reduction in DA-induced apoptosis, ROS production, oxidative stress, and improper synthesis of DA in neuronal cells 20. PD patients with a 40 bp deletion in the p337-376 allele of Exon 3, as well as in Exons 5 and 6, demonstrated a significant reduction in the expression of DAT 21. This objective can be achieved by modulating the activity of enzymes responsible for DAT post-translational modifications or by interfering with the interactions of its binding partners to either enhance or reduce DA reuptake. Extensive evidence has been accumulated to support the notion that the regulation of DAT-dependent DA uptake, DAT-mediated toxin accumulation, dysregulation of TH activity, and DAT signaling are all disrupted in PD 22. The capacity of the DAT to uptake DA is contingent upon its expression level in the plasma membrane. Furthermore, the membrane transport activity of DAT is associated with glycosylation modifications 23, 24. It has been observed that in the striatum of PD rat models treated with 6-hydroxydopamine and rDAT-HEK cell models exposed to 1-methyl-4-phenylpyridinium iodide, non-glycosylated DAT accumulates in the endoplasmic reticulum and Golgi apparatus, whereas glycosylated DAT is reduced in these organelles and on the plasma membrane. This suggests that the absence of glycosylation modification of DAT may hinder its trafficking to the plasma membrane in dopaminergic neurons 25. The dysfunctional regulation of the DAT can contribute to dopamine-related disorders, potentially leading to PD, schizophrenia, attention deficit hyperactivity disorder, and other central nervous system pathologies 26-28.

The proteins Hip1R, EPSIN 1, PICALM, and AP2μ2 interact with receptor proteins on the membrane to facilitate recognition and initiation of endocytic vesicle formation 29. The main interaction of Hip1R is with the actin cytoskeleton, thereby regulating the movement of endocytic vesicles 30; The role of EPSIN1 is to facilitate the recruitment and localization of clathrin on the cellular membrane 31; The PICALM gene has been implicated in the pathogenesis of neurodegenerative disorders, revising the regulation of clathrin-mediated endocytosis (CME) to exert influence on the recycling process of membrane proteins 32;.The AP2μ2 protein is a constituent of the AP-2 complex, which plays a crucial role in endocytosis by selectively recognizing specific membrane proteins 33. Protein kinase C (PKC) phosphorylates DAT, inducing conformational changes and promoting its internalization from the cell membrane to the cytoplasm, consequently reducing DA reuptake in the synaptic cleft 34. The degeneration of DAergic neurons in early PD patients may impact PKC activity, leading to disruption in the regulation of DAT endocytosis 35. This phenomenon can result in additional reductions in DA levels within the synaptic cleft, thereby exacerbating the condition 36. GRK2 facilitates the recruitment of β-arrestin to G protein-coupled receptors, such as D2Rs, by phosphorylating these receptors 37. The process also exerts an influence on the endocytosis of DAT and plays a pivotal role in the regulation of DA concentrations within the synaptic cleft 38. The function of DAT may become aberrant, resulting in impaired DA reuptake. This impairment could be associated with the degeneration of DAergic neurons and alterations in the cellular membrane structure 39. Some studies suggest that the endocytosis of DAT is impaired in PD patients, resulting in inadequate retrieval of DA from the synaptic cleft and further exacerbating DA deficiency. Dysfunction of DAT may enhance neuronal susceptibility to oxidative stress and toxic substances, thereby accelerating neuronal damage and death 40. The degeneration of these neurons not only leads to abnormalities in DA synthesis and release but may also involve disruptions in the endocytosis of DAT.

The apoptosis and demise of DAergic neurons constitute fundamental characteristics in the initiation and advancement of PD. The aforementioned pathological changes not only lead to a reduction in DA levels, resulting in the manifestation of typical motor symptoms, but also have the potential to further exacerbate the ongoing pathological process through intricate feedback mechanisms 41. The progression of DAergic neuron death involves the participation of biomarkers associated with apoptosis or cell death, such as caspases, Cytochrome c, Bax/Bcl-2, SNCA, p53 and so on 42. These biomarkers directly reflect the neurodegenerative processes of PD, thereby elucidating its pathological mechanisms and offering potential targets for early diagnosis and treatment. Consequently, molecular biomarkers associated with apoptosis hold significant research value in PD. Specifically, Caspase-3 serves as a pivotal executioner protease during the process of apoptosis 43. During the process of neuronal degeneration, caspase-3 is activated and triggers apoptosis. Therefore, measuring the activity or expression levels of caspase-3 may serve as a potential biomarker for detecting neuronal death in PD 44. The protein Apoptosis-inducing factor (AIF) plays a crucial role in the process of apoptosis by being released from mitochondria and translocating into the nucleus, where it triggers DNA fragmentation and subsequent cell death 45. The aberrant expression or altered subcellular distribution of AIF in PD may be associated with neuronal apoptosis 46. Oxidative stress is also a crucial mechanism contributing to neuronal death, and the identification of oxidative stress biomarkers can serve as an indicator of the extent of neuronal damage. 8-hydroxy-2'-deoxyguanosine (8-OHdG) serves as a reliable marker for DNA oxidative damage, and elevated levels of 8-OHdG in PD patients suggests the involvement of oxidative stress in neuronal apoptosis 47. Malondialdehyde (MDA) is a byproduct of lipid peroxidation, and elevated levels indicate the presence of oxidative stress and damage to cellular membranes 48. The alterations in MDA levels in PD may potentially indicate neuronal impairment 49. Inflammatory responses play a pivotal role in the pathological progression of the disease. The demise of DAergic neurons is frequently accompanied by neuroinflammation, and inflammatory markers hold potential for diagnostic and prognostic value in PD 50. Pro-inflammatory cytokines, such as IL-1β, TNF-α, and IL-6, are intricately associated with neuroinflammation and neuronal damage in PD. Inflammatory enzymes like cyclooxygenase-2 (COX-2) 51 and inducible nitric oxide synthase (iNOS) also play a significant role in the disease's inflammatory processes 52. The increased expression of COX-2 and iNOS in PD patients suggests that inflammation plays a contributory role in neuronal death. Biomarkers associated with apoptosis or demise of DAergic neurons are pivotal in neurodegeneration research, as they can facilitate early diagnosis, monitor disease progression, and evaluate treatment efficacy 53. The predictive or diagnostic capability of a single biomarker for PD is limited, but the integration of multiple biomarkers can enhance diagnostic accuracy and serve as a foundation for personalized treatment.

The vesicular monoamine transporter (VMAT) and DAT have distinct roles in the reuptake of DA, exhibiting significant variations in their functions and locations 54. The primary function of this transporter is to facilitate the translocation of DA from the cytoplasm into synaptic vesicles, thereby promoting its subsequent release. Conversely, the DAT is responsible for the reuptake of DA from the synaptic cleft back into neuronal cytoplasm, thus terminating signal transmission. Together, these transporters maintain DAergic homeostasis and ensure proper neurotransmission 55. VMAT is localized on the vesicular membrane of presynaptic nerve terminals, and its primary function is to facilitate the sequestration of cytoplasmic DA into synaptic vesicles for subsequent release during neurotransmission. This process involves VMAT employing a proton gradient-driven secondary active transport mechanism to translocate DA from the neuronal cytoplasm into the vesicles, which necessitates energy expenditure typically sustained by ATP-driven proton pumps that maintain the requisite proton gradient 56. DAT is situated on the plasma membrane of presynaptic neurons, directly facing the synaptic cleft. Its primary role entails reabsorbing unbound DA from the synaptic cleft and transporting it back into the cytoplasm of the presynaptic nerve terminal. This process aids in terminating DA signaling and facilitates recycling to some extent for subsequent reuse 57. The reuptake of DA by the VMAT occurs from the cytoplasm into the synaptic vesicles, serving to store and prepare DA for subsequent release. This process directly influences both the quantity of DA stored within these vehicles and the amount available for release. Conversely, the reuptake of DA by DAT takes place from the synaptic cleft back into the cytoplasm 58. The purpose is to remove DA from the synaptic cleft, terminate it signaling, and facilitate recycling. This process regulates the concentration of DA in the synaptic cleft, directly influencing the strength and duration of it signaling 59. The VMAT is a target for certain drugs (such as Ritalin and Amphetamine) in clinical settings, which enhance DA signaling by increasing the storage or release of it within vesicles 60. For example, reserpine, a DA inhibitor, can decrease the storage capacity of dopamine and result in reduced DA signaling. It is commonly used in the treatment of hypertension. DAT serves as a target for various drugs such as cocaine and specific antidepressants that enhance DA signaling within the synaptic cleft by inhibiting DAT and impeding its reuptake process. Abnormalities in clathrin-mediated endocytosis (CME) and clathrin-Independent endocytosis (CIE) are believed to be closely associated with neuronal dysfunction and death in PD. Endocytosis encompasses various types, including phagocytosis, pinocytosis, and receptor-mediated endocytosis, each serving distinct functions within specific contexts. It involves intricate membrane dynamics and is orchestrated by a collaborative network of proteins, constituting a vital pathway for cellular material exchange with the external environment. Notably, it plays a pivotal role in nutrient absorption, internalization of signaling molecules, and intercellular communication. Endocytosis is tightly regulated by the cell and involves multiple signaling pathways and protein interactions, ensuring selective uptake of necessary substances under appropriate conditions while preventing the entry of unnecessary or harmful materials. Clathrin-dependent endocytosis (CDE) is primarily utilized for internalizing specific ligands, such as hormones, transport proteins, and cell surface receptors. This mechanism exhibits high specificity and plays a crucial role in nutrient absorption, receptor downregulation, signal transduction, and viral entry into cells 61. The CIE pathways encompass a variety of mechanisms that do not rely on the formation of clathrin coats. These pathways are characterized by their diversity and are typically involved in the uptake of non-specific substances or play roles in processes such as signal transduction, immune response, and cell movement. While CIE exhibits lower selectivity compared to CDE, these pathways are crucial under certain physiological and pathological conditions. The main distinction lies in the fact that CDE strictly depends on clathrin and associated adaptor proteins, whereas CIE utilizes alternative proteins and pathways for endocytosis. CDE is highly specific and primarily involved in receptor-mediated endocytosis, whereas CIE exhibits lower specificity and is commonly utilized for broader uptake processes. CDE facilitates the formation of small vesicles coated with clathrin, while CIE can generate vesicles of varying sizes that may be uncoated or possess different types of coats. The main function of CDE lies in facilitating receptor-mediated uptake and downregulation, whereas CIE encompasses a wider range of biological processes including cell signaling, immune response, viral entry, and cell migration 62. SNCA is typically transported and degraded through CME or CIE. However, abnormal aggregation of SNCA can disrupt these endocytic pathways, leading to increased intracellular levels of SNCA and further promoting neuronal damage. Moreover, SNCA interacts with specific proteins in the CME pathway, potentially regulating its efficiency. The aberrant aggregation of SNCA may impair the normal function of CME, resulting in the accumulation of abnormal proteins within neurons and ultimately triggering cytotoxicity. The CIE pathway may also play a role in endocytosis and clearance of SNCA. The abnormal accumulation of SNCA can disrupt the functionality of the CIE pathway, leading to cellular metabolic dysregulation and mitochondrial dysfunction, thereby exacerbating neuronal death 63. The dysfunction of endocytic pathways, specifically in CME and CIE, may result in mitochondrial dysfunction, which is a crucial pathological process in PD. Mitochondrial damage can potentially impact intracellular energy metabolism by altering endocytic pathways, thereby leading to neuronal apoptosis or necrosis. Endocytosis plays a crucial role in immune cells, particularly microglia and astrocytes, and dysfunction in this process may exacerbate inflammatory responses, further compromising DAergic neurons and promoting the progression of PD. In normal conditions, endocytosis is vital for neuron regeneration and repair; however, disruption of these pathways can impede the neurons' ability to self-repair 64. The neuronal loss leading to PD is challenging to reverse. Given the intimate association between endocytosis and PD, pharmacological modulation of the CME and CIE pathways may emerge as potential therapeutic strategies. For instance, drugs augmenting the CME pathway could potentially restore normal SNCA transport and degradation, thereby decelerating disease progression. Enhancing SNCA clearance through both the CME and CIE pathways holds promise as a direction for PD treatment. The activation or regulation of these pathways may attenuate the toxic accumulation of SNCA and ameliorate neuronal damage. Clathrin-dependent and CIE pathways serve distinct roles in cellular material uptake and regulation, with their dysfunction closely associated with the pathogenesis of PD 65.

Epigenetic modifications are mechanisms of gene expression regulation that do not alter the DNA sequence, encompassing modifications to DNA and RNA, alterations to histones, and remodeling of chromatin structure. These modifications govern cellular function and disease progression by exerting influence over the transcriptional activity of genes 66. Epigenetic modifications play a crucial role in the initiation and progression of PD. DNA methylation is one of the most prevalent DNA modifications and serves as a pivotal regulatory mechanism for gene expression. The methylation status of the promoter region of the DAT gene directly impacts its expression levels. The upregulation of methylation typically leads to gene expression suppression, thereby resulting in a reduction of DAT expression on neuronal surfaces 67, 68. The DNA methylation status of specific genes undergoes alterations in PD. Consequently, this could result in dysregulation of dopamine levels within the synaptic cleft, leading to neuronal dysfunction and increased toxicity, ultimately promoting PD progression 69. RNA modifications are a recently discovered epigenetic mechanism, with N6-methyladenosine (m^6^A) being the predominant type of RNA modification. m^6^A modifications play a role in regulating the survival and function of DAergic neurons. These modifications can modulate the expression levels of PD-related genes, such as PINK1, which is essential for maintaining mitochondrial function. Aberrant m^6^A modification of the PINK1 gene may impact mitochondrial function, thereby promoting neuronal degeneration and driving PD progression 70, 71. Histone modifications encompass acetylation, methylation, phosphorylation, and ubiquitination. In the context of PD, alterations in histone acetylation levels are intricately associated with the progression of the disease. Augmented acetylation of histone H3 has the potential to facilitate the expression of specific neuroprotective genes, such as brain-derived neurotrophic factor (BDNF). BDNF plays a pivotal role in ensuring neuronal survival and maintaining optimal functionality; its diminished expression is linked to neuronal damage observed in PD. BDNF facilitates the maturation of DAergic neurons in the midbrain and positively modulates their maintenance and axonal outgrowth. Consequently, elucidating the mechanisms that regulate the BDNF signaling pathway may provide valuable insights into potential therapeutic strategies for neuropsychological disorders associated with dysregulated DAergic neurotransmission 72. By regulating the acetylation level of histone H3, the expression of BDNF can be modulated, thereby influencing the progression of PD. Increased levels of acetylation may enhance the expression and function of DAT, promoting its endocytosis and maintaining DA balance in the synaptic cleft. For instance, functional impairment of the Brg1 subunit within the SWI/SNF complex has been demonstrated in PD models, potentially leading to the suppression of specific neuroprotective genes and exacerbating neuronal death. Therefore, the dysfunction of chromatin remodeling complexes may impact the onset and progression of PD through modulation of chromatin structure 73. The studies on epigenetic modifications provide novel insights into the mechanisms underlying PD and may present potential targets for future therapeutic interventions.

Epigenetic regulation plays a pivotal role in the reprogramming and differentiation processes of induced pluripotent stem cells (iPSCs). By modulating mechanisms such as DNA methylation, histone modifications, non-coding RNAs, and chromatin remodeling, it is possible to exert influence on the maintenance of iPSC pluripotency as well as enhance the efficiency and functionality of DAergic neuron differentiation 74. In the investigation of reprogramming iPSCs into DAergic neurons, a range of epigenetic regulatory mechanisms have been validated in animal models and human-derived cell models. These specific mechanisms encompass DNA methylation, histone modifications, non-coding RNAs, and chromatin remodeling. The findings of these studies validate the pivotal role of epigenetic regulation in the differentiation process of iPSCs into DAergic neurons, thus providing robust scientific evidence for the potential application of cell replacement therapies in PD 75, 76.

The occurrence and progression of PD are directly influenced by epigenetic modifications, which regulate the endocytosis of DAT and the differentiation of DAergic neurons. The modifications not only regulate the expression and functionality of DAT but also exert an influence on the development and viability of DAergic neurons. Consequently, delving into the mechanisms underlying epigenetic modifications in PD provides novel insights and targets for both prevention and treatment strategies.

## Endocytosis of DAT is influenced by DNA modifications

The term "DNA modifications" are crucial in the regulation of gene activity, maintenance of genomic stability, and participation in diverse biological processes such as development, cellular differentiation, and disease occurrence 77. The process of DNA methylation predominantly takes place within the CpG islands located in gene promoter regions, thereby exerting an inhibitory effect on gene transcription 78. In the brains of PD patients with, aberrant methylation in specific gene regions may impact neuronal function, leading to neurodegenerative changes. This dysregulation can modulate the expression of genes associated with neuroinflammation, thereby influencing the immune response in PD and exacerbating neuronal damage. Genes involved in dopamine metabolism and neuroprotection may exhibit abnormal methylation patterns, resulting in apoptosis or dysfunction of DAergic neurons.

**Figure 1:**
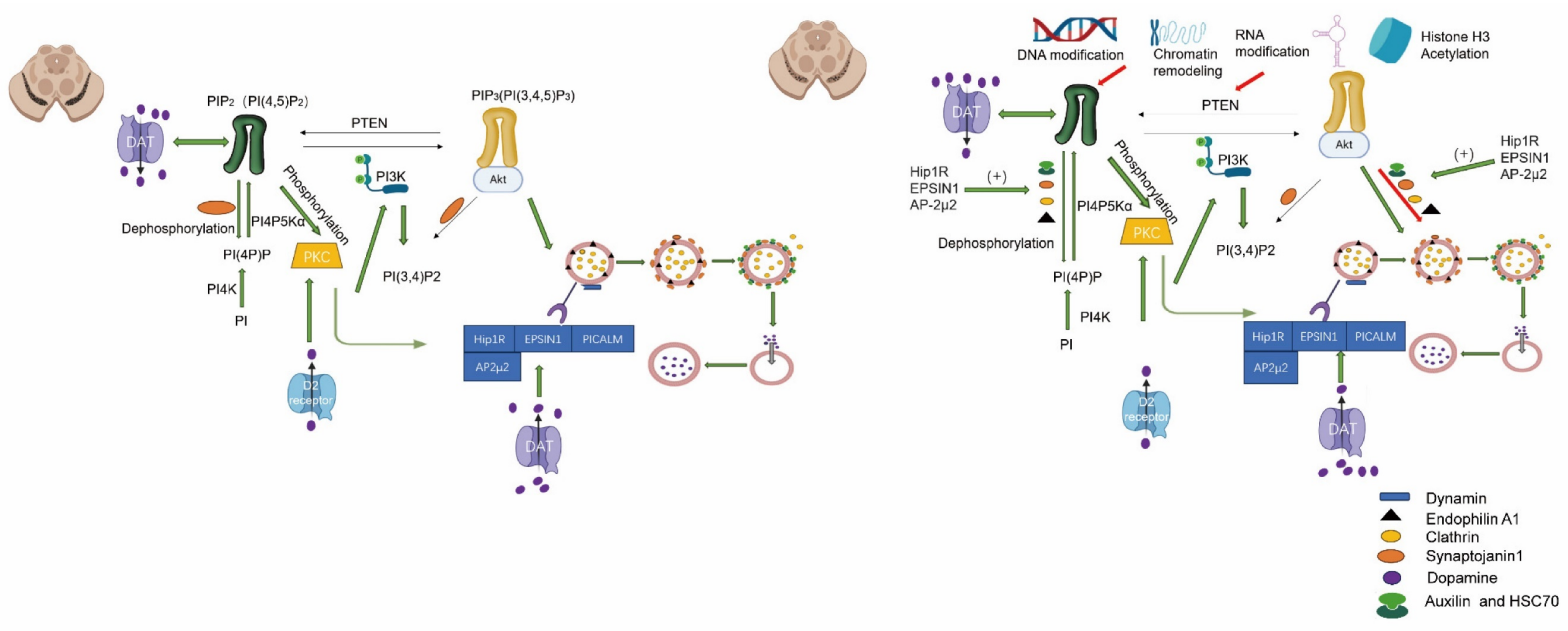
** The PKC and PI (4,5) P2 axis exert a significant influence on DAT endocytosis.** Elevated methylation of Hip1R, EPSIN1, and AP-2μ2 may alter the expression or activity of SYNJ1, Clathrin, HSC70 and Endophilin A1.

The occurrence of ^5^methylcytosine(^5^mC), is primarily observed in gene promoter regions and is commonly associated with gene silencing. Excessive methylation of differentiation-related gene promoters such as TH, DAT, and FOXA2 may impede their expression and hinder neuronal differentiation. Elevated methylation in the promoter regions of genes like Hip1R, EPSIN1, and AP-2μ2 may suppress the expression of Synaptojanin 1 (SYNJ1) and Auxilin, thereby impacting endocytic efficiency 79. SYNJ1 is a phosphoinositide phosphatase responsible for dephosphorylating PI (4,5) P_2_ to PI (4) P, thereby facilitating the removal of the clathrin coat during vesicle uncoating. Auxilin acts as a cofactor that binds to Hsc70 and recruits it to the clathrin coat, promoting ATP hydrolysis for disassembling clathrin 80, the presynaptic levels of DA are influenced by this, rendering neurons more vulnerable to degeneration and exacerbating neurodegenerative changes. By modulating the expression of genes associated with neuronal differentiation, it can exert an influence on the process of neuronal differentiation and enhance susceptibility to PD. Methylation generally acts as a suppressor of gene expression and underlies important biological phenomena such as genomic imprinting and X-chromosome inactivation 81. The DNA molecule wraps around histone proteins to form chromatin, and various post-translational modifications of histones can modulate the chromatin structure, thereby exerting an impact on gene expression. The formation of nucleosomes, which consist of DNA and histones, constitutes the fundamental units. Nucleosome remodeling refers to the process of altering the position or conformation of these units to facilitate access by regulatory factors involved in gene expression 82. The dynamic modifications play a pivotal role in cellular function and are intricately linked to numerous diseases, such as cancer and neurodegenerative disorders 83 (Figure 1, Table 1).

### Key enzymes governing the endocytosis of DAT during the process of DNA methylation

The enzymatic activity of DNMTs, which facilitate the addition of a methyl group to cytosine residues within the DNA molecule, thereby exerting an influence on gene expression 84. Although no single enzyme is definitively known to directly regulate DAT endocytosis, enzymes related to DAT endocytosis, cell signaling, and DNA methylation include DNMTs, PKC, epigenetic regulators such as TET proteins, D2Rs, and enzymes involved in DNA methylation. The most prevalent DNMTs, namely DNMT1, DNMT3A, and DNMT3B, exert their regulatory effects on gene expression through the methylation of specific gene promoter regions 85. Methylation regulation may indirectly impact DAT endocytosis by modulating the expression of signal molecules or related genes that govern DAT endocytosis, such as G-protein-coupled receptors or PKC. Although PKC itself is not a DNA methylation enzyme, it plays a pivotal role in the process of DAT endocytosis. The activity of PKC can be regulated by upstream signals, and the expression of these signaling pathways may sometimes be influenced by DNA methylation. PKC phosphorylates DAT, triggering its endocytosis and controlling the abundance of DAT molecules on the cellular surface. TET enzymes, including TET1, TET2, and TET3, play a crucial role in the oxidation of ^5^mC to ^5^hydroxymethylcytosine (^5^hmC), thereby contributing significantly to the process of DNA demethylation 86. TET enzymes may exert an influence on the expression of genes associated with DAT endocytosis by regulating their demethylation status, thereby indirectly impacting the process of DAT endocytosis. D2Rs could modulate both the function and endocytosis of DAT through signaling pathways. The gene expression of D2Rs can also be regulated by DNA methylation, which could potentially affect D2R's capacity to control DAT endocytosis 87.

### The DAT endocytosis process is regulated by the indirect pathway of DNA modifications

**Figure 2:**
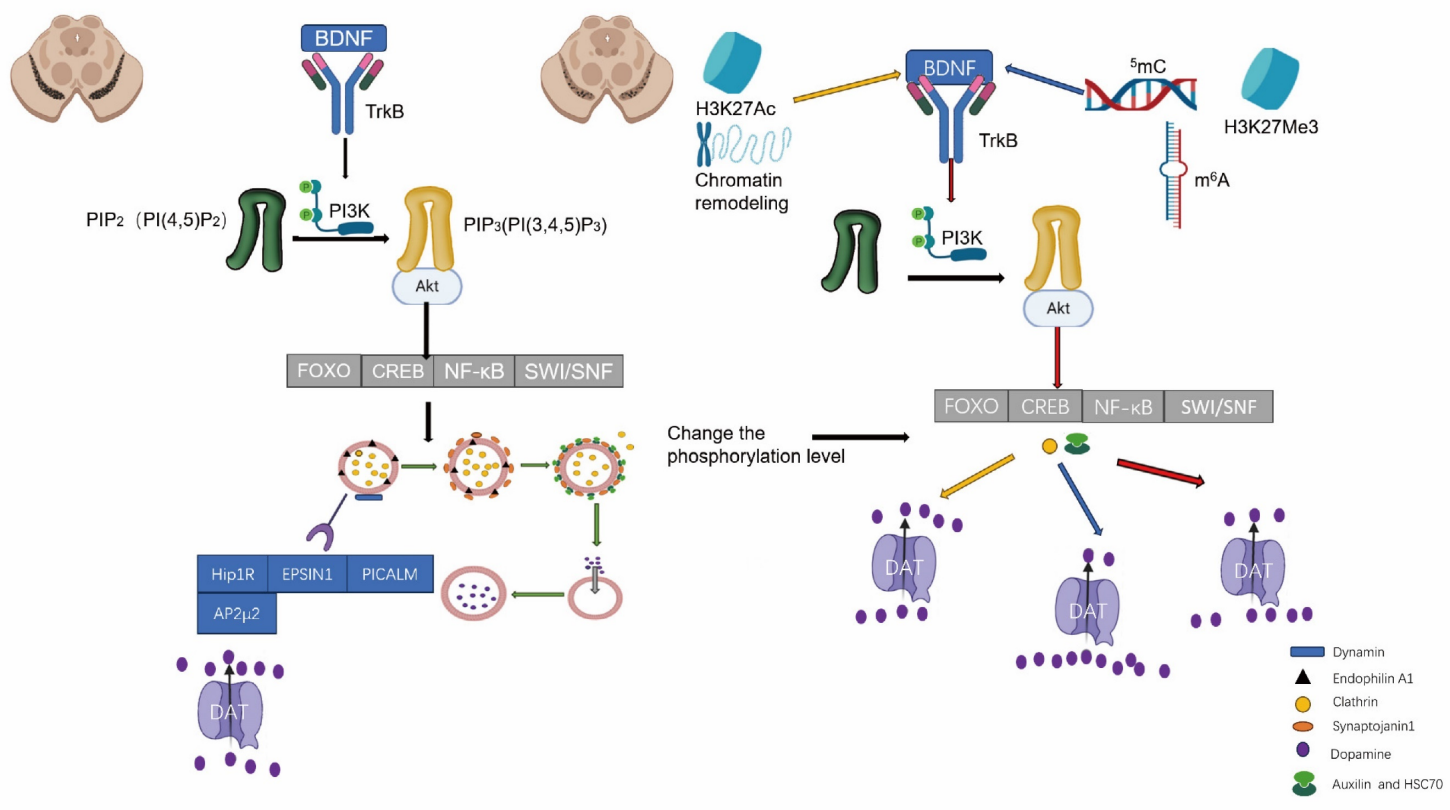
The regulation of DAT endocytosis is mediated by BDNF-TrkB signaling pathway.

Within the PKC signaling pathway, PKC serves as a pivotal regulatory factor for DAT endocytosis by facilitating its internalization from the cell membrane through phosphorylation 88. The activity of PKC can be modulated by DNA methylation through the suppression or activation of upstream regulatory molecules in the PKC signaling pathway 89. The state of DNA methylation can impact on the synthesis of phosphatidylinositol (PIP_2_) or other lipid molecules, which govern the activation of PKC 90. The PI (4,5) P_2_ molecule plays a crucial role in the regulation of CME, and CME relies on it for DAT internalization. Specifically, PI (4,5) P_2_ facilitates DAT endocytosis by recruiting various proteins involved in endocytosis, including the AP2 complex, adaptor proteins, and dynamin. Moreover, it regulates membrane curvature and promotes the formation of endocytic vesicles. The interaction between PI (4,5) P_2_ and specific transmembrane proteins directly regulates their cellular distribution and endocytosis on the plasma membrane. In the case of DAT, PI (4,5) P_2_ may modulate its functionality by binding to either its transmembrane domain or intracellular tail, or by influencing proteins involved in DAT endocytosis such as PKC or cytoskeletal components like β-actin. The dynamic distribution and metabolism of PI (4,5) P_2_ on the cell membrane can enhance vesicle formation associated with DAT endocytosis. Conversely, the action of phosphatases such as SYNJ1 can inhibit the process of DAT endocytosis. While the regulatory role of PI (4,5) P_2_ in DAT endocytosis is well established, some studies suggest that DAT activity may, in turn, influence its local concentration. For instance, the transport activity of DAT can modulate membrane potential and subsequently impact the function of phospholipases (such as PLC), or enzymes involved in phosphatase metabolism. The use of drugs such as amphetamine can indirectly impact the production and degradation of PI (4,5) P_2_. In the D2R signaling pathway, the activation of G protein-coupled signaling pathways by the D2 receptor regulates the internalization of DAT. The expression of the D2 receptor gene may be modulated by DNA methylation, thereby influencing D2R-mediated signaling pathways and subsequently controlling DAT internalization 91. Epigenetic regulation, including histone modification and DNA methylation, can modulate multiple molecular pathways associated with DAT internalization in neurons. The acetylation or deacetylation of histones alters chromatin accessibility, thereby impacting gene expression. DNA methylation collaborates with histone modification to influence DAT internalization by either inhibiting or activating the expression of specific signaling molecules. The internalization of DAT is dependent on the dynamic restructuring of the cytoskeleton, specifically the microtubule and microfilament systems. Furthermore, the expression of genes related to the cytoskeleton, such as actin and microtubule-associated proteins, modulates the interaction between DAT and the cytoskeleton, thereby regulating the process of internalization 92. The stress response modulates the internalization and functionality of DAT by activating a cascade of signaling pathways. DNA methylation can impact on the expression of genes associated with the hypothalamic-pituitary-adrenal axis, thereby altering cortisol levels, which are pivotal stress hormones. These hormones regulate DAT function through signaling pathways, subsequently influencing the process of internalization 93. In an oxidative stress environment, DNA methylation may regulate the expression of genes encoding antioxidant proteins, thereby influencing intracellular DA metabolism and modifying the processes of DAT expression and internalization. Non-coding RNAs, particularly miRNAs and lncRNAs, play crucial roles in regulating DNA methylation by indirectly modulating the expression of target genes associated with DAT internalization. Certain miRNAs can modulate the function of DAT by regulating the expression of DAT itself or its associated proteins. For instance, DNA methylation may regulate certain miRNA genes involved in the internalization process, thereby altering their expression levels. Long non-coding RNAs can indirectly regulate genes associated with DAT internalization through modulation of DNA methylation status or interaction with epigenetic regulators. Growth factor signaling pathways, such as BDNF, play a pivotal role in governing neuronal function and neuroplasticity 94. DNA methylation indirectly influences DAT internalization by modulating the expression of growth factors and their receptors. The TrkB receptor mediates BDNF's regulation of DAT function and internalization. The DNA methylation status can modulate the expression of the BDNF gene, thereby altering the regulatory impact of BDNF signaling on DAT internalization 95. The ubiquitin-proteasome system (UPS) regulates the internalization and recycling of DAT through protein degradation. DNA methylation can modulate the expression of genes associated with UPS, thereby indirectly influencing DAT internalization. By regulating the expression of E3 ubiquitin ligases, DNA methylation affects the ubiquitination and internalization of DAT 12. The regulation of multiple signaling pathways by DNA modifications indirectly influences the process of DAT internalization. These pathways exert their effects on the expression or function of genes and proteins associated with DAT internalization, thereby impacting the dynamics of this cellular process (Figure 2).

### The internalization of DAT is modulated by small molecule inhibitors through DNA modifications

The expression of relevant genes during DNA modification can be indirectly influenced by small molecule inhibitors, thereby impacting the function and internalization of the DAT. The drug 5-Aza-2'-deoxycytidine (5-Aza-dC) inhibits DNMTs, thereby impeding the maintenance of DNA methylation and subsequently reinstating the expression of genes that have been epigenetically silenced 96, By alleviating the transcriptional repression of genes associated with the regulation of DAT internalization, 5-Aza-dC may indirectly modulate the process of DAT internalization. Decitabine, a widely used DNA methylation inhibitor, can enhance the expression of regulatory genes involved in internalization by reducing their methylation levels, thereby influencing the functionality and internalization dynamics of DAT 97. The alteration of gene methylation status induced by these inhibitors may lead to an increase in the expression of associated proteins (such as endocytosis regulators or signaling molecules) that are involved in endocytosis and DAT function, thereby indirectly influencing the rate or stability of DAT internalization. TET enzymes play a crucial role in the conversion of ^5^mC to ^5^hmC, thereby facilitating DNA demethylation. Inhibition of TET enzymes can effectively impede DNA demethylation process and maintain specific genes in a repressed state, ultimately regulating genes associated with DAT internalization. By modulating the hydroxy methylation status of DNA 98, The expression of genes related to DA signaling may be regulated by TET inhibitors 99, endocytosis and recycling indirectly impact the function and efficiency of DAT internalization 100.

The inhibition of key enzymes such as DNMTs, histone deacetylases (HDACs), and histone methyltransferases (HMTs) can induce alterations in chromatin states or the states of gene silencing/activation 101. The modulation of DAT function and internalization processes is achieved by affecting the expression of proteins involved in endocytosis, vesicular trafficking, and signal transduction. Consequently, the application of these small molecule inhibitors not only serves as valuable tools for fundamental research but also holds potential therapeutic strategies for DA-related diseases such as PD and schizophrenia.

## The function of DAT endocytosis is influenced by RNA modifications

RNA modifications significantly impact gene expression, neuronal survival, and antioxidant responses, thereby contributing to the initiation and progression of this disorder. Aberrant RNA modifications can detrimentally affect DAergic neuron health and consequently facilitate PD advancement 102. The phenomenon has been extensively investigated in diverse biological processes and pathological conditions in recent years.

### Key enzymes implicated in RNA modifications that impact the process of DAT endocytosis

The expression of DAT may also be regulated by m^6^A modification. The m^6^A methyltransferase complex (referred to as "writer" enzymes), METTL3 and METTL14, comprises these two proteins that serve as the primary methyltransferases responsible for m^6^A modification. The addition of methyl groups to mRNA adenosine can be facilitated by the METTL3/METTL14 complex. This complex has the potential to regulate mRNA modifications, thereby influencing the expression levels of proteins associated with DAT endocytosis 103. The m^6^A demethylases, FTO and ALKBH5, are enzymes referred to as "eraser" enzymes that possess the ability to eliminate m^6^A modifications from mRNA molecules, thereby impacting both the stability and translation of these mRNA molecules 104. The modulation of mRNA encoding proteins involved in endocytosis allows for an indirect regulation of DAT endocytosis. If m^6^A modification enhances the stability and translation of mRNA associated with genes related to DAT endocytosis, such as those involved in vesicle formation and cytoskeletal reorganization, it may speed up DAT endocytosis. Conversely, demethylation can lead to a reduction in the expression of these genes, thereby attenuating the incidence of DAT endocytosis. Additionally, RNA editing represents an alternative mechanism for RNA modification, wherein ADAR enzymes catalyze the conversion of adenosine (A) to inosine (I) within RNA molecules 105. The process of mRNA translation, stability, and protein function can be influenced by this editing. ADAR exerts its effects on genes that encode proteins involved in DAT endocytosis through RNA editing, thereby modifying the function or expression of these proteins and subsequently impacting the endocytosis process. ADAR1 and ADAR2, as the primary adenosine deaminases, are responsible for converting A to I, resulting in alterations in mRNA splicing or translation efficiency. Through modification of mRNA sequences associated with various proteins related to DAT endocytosis, ADAR influences the function or translation efficiency of these proteins, thus regulating DAT endocytosis. If ADAR edits specific proteins involved in endocytosis to generate novel isoforms, it could potentially alter vesicle formation and the efficiency of endocytosis, thereby impacting DAT endocytosis. Pseudouridine is a prevalent RNA modification that may indirectly influence the expression of genes associated with DAT endocytosis by enhancing mRNA stability and translation efficiency. The pseudouridine synthase enzyme family is responsible for converting uridine (U) into pseudouridine 106. If pseudouridine modification occurs on the mRNA of genes involved in DAT endocytosis (such as those encoding cytoskeletal proteins or key regulators in the endocytic pathway), it has the potential to upregulate the expression of these proteins, thereby enhancing the process of DAT endocytosis. The ^5^methylcytosine modification is a prevalent type of modification observed in tRNA, rRNA, and mRNA, which exerts regulatory effects on mRNA stability, translation efficiency, and ribosome biogenesis. NOP2/Sun RNA methyltransferase family member 2(NUSN2) is a ^5^methylcytosine methyltransferase responsible for catalyzing cytosine methylation into ^5^methylcytosine 107. The enzyme DNMT2 primarily methylates ^5^methylcytosine in tRNA molecules, but it may also exert an influence on mRNA modifications, thereby regulating the process of protein synthesis 108. The m^5^C modification can modulate the stability or translation efficiency of mRNA associated with DAT endocytosis genes, thereby impacting the protein output encoded by these genes. If m^5^C modification upregulates the expression of proteins involved in endocytosis, signal transduction, or cytoskeleton organization, it may facilitate DAT endocytosis. RNA-binding proteins (RBPs) engaged in mRNA splicing, transport, and translation regulation are frequently methylated on arginine residues to ensure their functional integrity. Changes in arginine methylation can regulate the activity of RBPs involved in DAT endocytosis. Protein arginine methyltransferases (PRMTs) are responsible for enzymatically adding methyl groups to arginine residues, thereby modulating the function of RBPs. Through this regulatory mechanism, PRMTs exert influence on the functionality of RBPs 109, The aforementioned phenomenon exerts an influence on the post-transcriptional processing and translation of mRNA associated with DAT endocytosis, thereby exerting an impact on the dynamics of DAT endocytosis 110, 111.

The key enzymes involved in RNA modification that regulate DAT endocytosis also control the mRNA modifications of genes associated with DAT endocytosis, thereby influencing their translation, stability, or splicing. Consequently, this regulatory mechanism impacts the synthesis and function of proteins involved in DAT endocytosis, ultimately affecting the process of DAT endocytosis indirectly.

### The indirect pathway governing RNA modifications regulates the process of DAT endocytosis

RNA modifications can indirectly regulate DAT endocytosis by influencing the expression or functionality of signaling molecules or receptors associated with this process. These signaling pathways may encompass key regulators of endocytosis such as GPCRs, PKC, and small GTPases. The expression of these proteins can be influenced by m^6^A, which regulates the mRNA stability or translation efficiency of signaling proteins. Given the close relationship between GPCRs and endocytosis, m^6^A may indirectly regulate DAT endocytosis by modulating the translation of D2R mRNA. D2R activation modulates DAT membrane expression through downstream signaling pathways. Activation of PKC can facilitate the endocytosis of DAT, while m^6^A modifications regulate the translation of PKC subunits, thereby influencing the endocytic process. RNA editing can modify the activity of signaling proteins involved in endocytosis (such as Ras family small GTPases) by editing their mRNA, consequently impacting vesicle formation and endocytic pathways, indirectly regulating DAT endocytosis. Pseudouridine modifications have the potential to enhance the translation of specific proteins involved in signaling pathways, particularly those associated with membrane remodeling and endocytosis, thereby exerting an impact on downstream DAT endocytosis. RNA modifications can exert an influence on the efficiency of protein translation associated with DAT endocytosis, thereby modulating the process of endocytosis. These proteins encompass key regulators involved in pivotal steps such as vesicle formation, cytoskeletal reorganization, and ubiquitination. m^6^A modifications have the potential to alter the synthesis levels of specific proteins implicated in vesicle formation (e.g., Clathrin and AP-2 complex subunits) by modulating mRNA stability, consequently impacting DAT endocytosis efficiency indirectly. For instance, m^6^A modifications may enhance the expression of key regulatory proteins involved in endocytosis, thereby augmenting DAT endocytosis capacity. By increasing the translation of cytoskeletal-related proteins (e.g., actin and tubulin), pseudo uridine modifications may expedite the dynamic reorganization of the cytoskeleton, indirectly facilitating DAT endocytosis 112. The mRNA stability of ubiquitination regulatory proteins in the endocytic pathway can be regulated by m^5^C modifications, thereby modulating the ubiquitination of DAT, which plays a crucial role in endocytosis. RBPs and translation factors serve as pivotal regulators of RNA modifications, exerting influence on post-transcriptional regulation and protein translation through RNA modifications such as m^6^A and m^5^C 113. The function of these proteins can be altered by RNA modifications, thereby indirectly impacting the synthesis of proteins associated with DAT endocytosis. m^6^A-binding proteins, such as the YTHDF family, specifically bind to regions with m^6^A modifications and regulate the translation and degradation processes of target mRNAs. The YTHDF proteins could interact with specific mRNAs that are associated with endocytosis, thereby exerting an influence on the translation efficiency of these proteins and ultimately regulating DAT endocytosis 114. The methylation of arginine residues on certain RBPs can modulate their mRNA recognition capacity, thereby impacting the stability and translation efficiency of endocytosis-related mRNAs. NSUN2 enhances the translational regulatory potential of these proteins by promoting m^5^C modifications on the mRNA molecules encoding RBPs, thus exerting influence over the expression of genes associated with endocytosis 115, 116. The function of RBPs, potentially involved in mRNA transport, splicing, or translation related to endocytosis, is regulated by PRMT1, an arginine methyltransferase through methylation 117. After endocytosis, DAT is typically degraded through the ubiquitination and proteasomal pathway. RNA modifications can indirectly influence the degradation or recycling of DAT post-endocytosis by regulating the expression of enzymes involved in ubiquitination. m^6^A may regulate the expression or activity of ubiquitin ligases, thereby impacting the ubiquitination process of DAT. By upregulating the expression of ubiquitination-associated proteins, m^6^A modifications can enhance the post-endocytic degradation of DAT, while downregulation of these proteins may facilitate the recycling process of DAT back to the cellular membrane. Removal of m^6^A from ubiquitination-related proteins could potentially decrease their translation, thereby impacting the post-endocytic fate of DAT and determining whether it undergoes degradation or recycling to the membrane. The cell's ability to regulate DAT endocytosis can be influenced by RNA modifications, which modulate pathways indirectly related to endocytosis, such as stress responses and metabolic pathways. Specific signaling pathways activated during cellular stress can alter the patterns of DAT endocytosis, and these pathways are regulated by RNA modifications. m^6^A modifications have the potential to impact cellular metabolic levels, thereby influencing ATP-dependent processes associated with endocytosis. Alterations in the expression of metabolic enzymes may indirectly affect the energy supply required for DAT endocytosis. During stress conditions, RNA modifications could potentially regulate the translation of stress response genes and indirectly influence the speed and efficiency of DAT endocytosis.

The process of DAT endocytosis is regulated by RNA modifications through various indirect pathways, exerting their influence on key proteins and signaling molecules involved in the endocytosis process to effectively modulate the quantity and function of DAT on the cell membrane.

### The DAT endocytosis process is targeted by small molecule inhibitors of RNA modifications

Small molecule inhibitors can indirectly regulate the function and endocytosis of the DAT by impacting chemical modifications such as m^6^A and m^5^C. These inhibitors typically modulate RNA translation, stability, and post-transcriptional regulation, thereby influencing the synthesis of genes and proteins associated with DAT expression or endocytosis. The METTL3 inhibitor, STM2457, effectively reduces m^6^A on mRNA by inhibiting the activity of METTL3, potentially impacting the synthesis of proteins involved in DAT endocytosis 118. The FTO inhibitor DAA functions as a demethylase responsible for the removal of m^6^A modifications from mRNA. By inhibiting FTO, the stability of m^6^A modifications is increased, leading to alterations in the expression of specific genes associated with DAT endocytosis 119. m^6^A modifications exert an impact on the stability of mRNA and the efficiency of translation for signaling proteins as well as endocytosis-related proteins, including Clathrin and AP-2. By inhibiting METTL3 or FTO, it is possible to indirectly influence DAT endocytosis and recycling through modulation of the expression levels of these proteins. For instance, the inhibition of FTO can elevate m^6^A levels on genes associated with DAT recycling, thereby leading to a reduction in DAT endocytosis. Inhibiting m^6^A can attenuate the stability of DAT mRNA, thereby diminishing its translation and consequently impacting its expression on the cellular membrane. If the stability of DAT mRNA decreases, it may lead to a reduction in membrane expression of DAT, which could potentially affect DA reuptake. Additionally, m^5^C modifications also exert significant regulatory effects on tRNA and mRNA by influencing their stability, translational efficiency, and cytoplasmic transport. NSUN2 serves as the primary methyltransferase responsible for catalyzing cytosine methylation to cytosine 115. NSUN2 inhibitors decrease the mRNA stability of proteins involved in the endocytosis process, such as Dynamin and Rab GTPase, thereby reducing their synthesis and potentially impeding the rate of DAT endocytosis. Inhibiting m^5^C may result in decreased mRNA stability of genes associated with DAT endocytosis, leading to reduced protein levels and subsequently impacting the efficiency of DAT endocytosis.

By interfering with the process of RNA editing, ADAR inhibitors have the potential to modulate the function or expression of genes associated with DAT endocytosis. Furthermore, RNA editing could influence splice isoforms or translation efficiency of specific proteins involved in endocytosis, thereby regulating DAT endocytosis. ADAR inhibitors may modulate the protein expression necessary for DAT endocytosis by reducing gene editing, thereby indirectly regulating the stability of DAT membrane. RNA editing plays a crucial role in the regulation of small GTPase signaling pathways. Inhibiting ADAR can indirectly impact DAT endocytosis by altering the editing status of signaling proteins related to endocytosis. RBPs are pivotal regulators of post-modification RNA function, influencing mRNA fate through interactions with modifications such as m^6^A and m^5^C. The YTHDF family proteins specifically recognize m^6^A modifications and regulate mRNA stability and degradation. Suppression of their function can potentially modify the translation efficiency of mRNAs associated with DAT endocytosis, thereby impacting DAT functionality. By inhibiting YTHDF proteins, the translation efficiency of genes related to DAT endocytosis can be modulated. If the translation of these genes is decreased or dysregulated, it may lead to a deceleration in DAT endocytosis or disruption in its recycling process. Additionally, YTHDF proteins play a role in regulating mRNA degradation rate. Through modulation of the degradation rate of m^6^A-marked mRNAs, inhibitors targeting YTHDF can indirectly influence the expression levels of proteins associated with DAT function and thereby impact DAT endocytosis. The ubiquitination pathway is regulated by RNA modifications, which can indirectly impact DAT endocytosis and degradation. m^6^A or other modifications modulate the expression of genes associated with protein degradation, thereby influencing the fate of DAT. The expression of ubiquitin ligases (E3) and deubiquitinating enzymes, which regulate the ubiquitination state of DAT and determine its degradation or recycling after endocytosis, can be influenced by small molecule inhibitors targeting such modifications.

In the process of RNA modifications, genes or proteins synthesis associated with DAT expression, endocytosis, and recycling are indirectly regulated. The inhibition of these modifications or the proteins involved in which can impact signaling pathways, endocytic proteins, and ubiquitination mechanisms that play a role in endocytosis, thereby regulating the function and stability of DAT on neuronal membranes.

## The endocytosis of DAT is influenced by alterations in histone signaling

Changes in histone signaling profoundly impact the pathological processes of PD by modulating the expression of genes associated with neuroprotection, inflammatory responses, and stress responses. Aberrant histone modifications can contribute to the degeneration and demise of DAergic neurons, thereby exacerbating their progression. Specific sites on histone H3 in PD patients display abnormal methylation patterns that may disrupt the expression of relevant genes 120. The study of nucleosome remodeling has also focused on its role in the regulation of gene expression. Dysregulation of PD-related genes may occur due to abnormal expression or dysfunction of specific nucleosome remodeling complexes. The crucial role of DNA modifications in PD necessitates the exploration of epigenetic regulators, such as inhibitors targeting DNA methylation and histone deacetylase, as potential therapeutic strategies 121. The process of acetylation typically occurs on lysine residues, resulting in a reduction in the affinity between histones and DNA, thereby inducing a more relaxed chromatin structure that facilitates gene transcription activity. Histone acetyl transferases (HATs) are responsible for adding acetyl groups, while HDACs are responsible for removing these groups 122, 123. The methylation process can occur on lysine or arginine residues, exerting either a promotive or inhibitory effect on gene expression. The specific impact is determined by the modification site and the degree of methylation (mono-, di-, or tri-methylation). HMTs are responsible for adding methyl groups, while histone demethylases remove them. Phosphorylation commonly occurs on serine, threonine, and tyrosine residues and primarily governs DNA damage repair and cell cycle regulation. Ubiquitination entails the addition of ubiquitin proteins to histones; this modification predominantly facilitates protein degradation but also exerts control over gene expression on histones 122, 124. The transcriptional activity of genes is directly influenced by histone modifications, which alter the chromatin's state either open or closed. Acetylation and methylation at specific sites can induce chromatin relaxation and enhance gene expression, whereas methylation at other sites can condense chromatin and suppress gene expression. These modifications determine the temporal expression of genes. They play a pivotal role not only in development but also in the cellular response to environmental changes. Modifications such as phosphorylation can recruit DNA repair proteins, thereby facilitating the repair of DNA damage, which is crucial for maintaining genomic stability. Histone modifications can impact cell differentiation, proliferation, and apoptosis, thus regulating cell fate determinations. Common alterations in histone signaling observed in cancer include heightened activity of HDACs, leading to the suppression of tumor suppressor genes 125, 126. Additionally, aberrant activity of specific HMTs and demethylases is also linked to the development of tumors. In neurodegenerative disorders like AD and PD, dysregulation of histone signals may contribute to abnormal gene expression in neurons, thereby exacerbating neuronal injury and death. The role of histone signal variations in cardiovascular diseases is also gaining increasing attention, particularly with regards to the close association between histone deacetylation and the regulation of genes associated with atherosclerosis. Given the crucial role of aberrant histone signaling in various pathological conditions, targeting histone modifications has emerged as a prominent area of research. For instance, HDAC inhibitors are currently employed for the treatment of specific cancer types 127. Additionally, there is active development of drugs that target histone methylation and phosphorylation. Alterations in histone signaling have profound impacts on cellular biological functions through the regulation of gene expression, chromatin structure, and cell fate. These changes are not only associated with normal physiological processes but also play a pivotal role in the pathogenesis of numerous diseases.

Histone signaling changes refer to the dynamic alterations of histone modifications, such as acetylation, methylation, phosphorylation, and ubiquitination, within cells. These modifications impact chromatin structure and gene expression, thereby regulating various biological processes in cells. Key signals include the activation marker H3K27Ac and the repression marker H3K27Me3. The activation signals are associated with the activation of genes and could enhance gene expression, such as Bcl-2, as well as promote the expression of differentiation marker genes like TH, DAT, and FOXA2, thereby facilitating neuronal differentiation. H3K27Ac is enriched in the promoter regions of genes like Hip1R, EPSIN1, and AP-2μ2, which leads to an enhancement in receptor expression. An upregulation of these receptors may enhance the endocytic activity of DAT, thereby impacting the recycling process of synaptic vesicles and dopamine reuptake. Repression signals are associated with gene silencing and can inhibit the expression of pro-apoptotic genes such as p53. Aberrant levels of H3K27Me3 may further diminish neuronal survival and exacerbate symptoms related to Parkinson's disease. The promoter regions of genes such as tyrosine hydroxylation (TH) and DAT display an enrichment in H3K27Me3, resulting in the suppression of their expression and hindering neuronal differentiation. Increased levels of H3K27Me3 may impede the expression of specific receptor genes, potentially compromising the efficiency of DAT endocytic processes and affecting the stability of presynaptic dopamine levels. Abnormal elevations in H3K27Me3 can lead to impaired neuronal differentiation, reducing the generation of functional neurons and further exacerbating PD symptoms 128.

### Key enzymes influencing the endocytosis of DAT via histone signal variations

In the process of histone signaling changes, key enzymes that impact DAT endocytosis primarily modulate chromatin state and gene expression by regulating histone acetylation, deacetylation, methylation, and demethylation. These enzymes can indirectly or directly influence DAT function by modifying the expression of genes associated with endocytosis, internalization, and recycling. The addition of acetyl groups to lysine residues on histones by HATs promotes gene transcription, resulting in a more accessible chromatin structure. CBP/p300, as important HATs, maintain an open state of chromatin through the acetylation of histones H3 and H4, thereby facilitating the expression of genes associated with DAT endocytosis. For instance, they may enhance the expression of endocytosis-regulating proteins such as Clathrin and AP-2 complexes, thereby augmenting the velocity and efficacy of DAT endocytosis. CBP/p300 could potentially facilitate the formation of endosomes and regulate endocytosis by modulating the crucial gene expression involved in these processes, ultimately promoting DAT endocytosis and its recycling. HDACs repress gene transcription by deacetylating histones, resulting in more compact chromatin structure. The enzymatic activity of these proteins can impede the expression of genes involved in regulating DAT endocytosis, thereby impacting the process of DAT internalization. The HDAC1/2 members of the HDAC family exert chromatin compaction by deacetylating histones, thereby suppressing the expression of genes involved in DAT endocytosis regulation. Reduced HDAC activity may enhance the expression of DAT-related proteins, thereby promoting endocytosis. SIRT1, an NAD^+^ dependent deacetylase involved in various cellular processes, can modulate key proteins in the endocytosis process to influence DAT endocytosis and recycling. HDAC inhibitors (such as Trichostatin A, TSA) can upregulate genes related to DAT endocytosis regulation, including those involved in endosomes, Clathrin, and Dynamin, by increasing histone acetylation levels and facilitating chromatin accessibility. The activity state of chromatin is regulated by HMTs through the addition of methyl groups to lysine or arginine residues on histones. Methylation can either facilitate gene activation (e.g., H3K4 methylation) or hinder gene expression (e.g., H3K27 methylation). SETD1A/B members possess the ability to catalyze histone H3K4 methylation, which is typically associated with active chromatin and gene activation. By upregulating the expression of genes involved in regulating endocytosis, they may indirectly modulate DAT endocytosis. EZH2, a component of the Polycomb Repressive Complex 2 (PRC2) complex responsible for H3K27Me3 modification, is typically associated with transcriptional repression. EZH2 could potentially enhance DAT endocytosis and recycling by reducing inhibitory methylation marks, thereby alleviating suppression of genes associated with the endocytic process. Through the upregulation of gene expressions involved in regulating endocytosis, they can indirectly influence DAT endocytosis. EZH2, a constituent of the PRC2 complex accountable for H3K27Me3 modification, is commonly linked to transcriptional repression. The inhibition of EZH2 has the potential to boost DAT endocytosis and recycling by diminishing inhibitory methylation marks, thus relieving suppression on genes related to the process of internalization. Methyltransferases regulate the expression of genes associated with DAT endocytosis, including proteins involved in endosome formation and recycling. EZH2 inhibitors can enhance the expression of genes related to DAT endocytosis by reducing H3K27Me3 marks. Histone lysine demethylases (KDMs) modulate chromatin accessibility and gene expression by demethylating histones. Demethylation can either activate or suppress gene expression, depending on the specific methylation marks present. KDM5B functions as a demethylase responsible for removing H3K4Me3, which is a mark typically associated with gene activation. By reducing the presence of active gene marks, KDM5B may impede the expression of genes involved in DAT endocytosis regulation. Members of the KDM6 family possess the ability to eliminate H3K27Me3, a mark commonly linked to gene silencing 129. By removing this inhibitory mark, KDM6 facilitates the activation of genes associated with DAT endocytosis. KDM6 enhances the expression of proteins involved in regulating endocytosis by eliminating H3K27Me3, thereby alleviating the suppression of genes linked to endocytosis and recycling processes. The modulation of these regulatory pathways by KDM inhibitors can regulate DAT endocytosis and cell surface expression.

Key enzymes such as HATs, HDACs, HMTs, and KDMs regulate the chromatin's state of openness or compactness, thereby influencing the expression of genes associated with DAT endocytosis. These enzymes can impact the expression of proteins involved in regulating endocytosis, forming CME, and recycling processes, consequently affecting DAT endocytosis and trafficking.

### Regulation of DAT endocytosis in histone signaling via the indirect pathway

The indirect pathway modulates DAT endocytosis through its regulation of gene expression, signal transduction, protein translation, and intracellular molecular mechanisms. Histone modifications induce changes in chromatin structure that impact the expression of genes involved in endocytosis and recycling processes. These genes may not directly govern DAT function but rather exert their influence on proteins implicated in the endocytic machinery. Histone acetylation or methylation activates genes associated with endocytosis (such as Clathrin, Dynamin, and AP-2), thereby enhancing their expression and indirectly facilitating DAT endocytosis. The expression of ubiquitination and deubiquitylation-related genes, which regulate DAT degradation or recycling and thus control endocytosis, is also influenced by changes in chromatin structure. EZH2, a member of the PRC2 complex, mediates gene silencing involved in endocytosis and vesicle formation through histone H3K27 methylation. Inhibiting EZH2 can activate these genes, thereby facilitating DAT endocytosis and recycling. Histone modifications indirectly regulate DAT endocytosis by modulating key molecules involved in signal transduction pathways, including kinases, phosphatases, and transcription factors that are crucial for the endocytic process. HATs and HDACs impact gene expressions in pathways such as PI3K-Akt and MAPK, which govern endocytosis. The activated MAPK pathway can enhance DAT endocytosis by promoting the phosphorylation of proteins related to endocytosis (such as Clathrin). HDAC inhibitors facilitate chromatin accessibility and activate upstream regulatory genes in the PI3K-Akt pathway, thereby enhancing Akt activity and promoting DAT endocytosis through signal transduction. Histone modifications also regulate the expression of genes involved in the ubiquitination pathway, thereby indirectly impacting DAT endocytosis, degradation, or recycling processes. The fate of DAT after endocytosis is determined by its ubiquitination status, which dictates whether it undergoes recycling or degradation. Histone acetylation or deacetylation controls the expression of E3 ubiquitin ligases, which are pivotal regulators in both endocytosis and degradation pathways. E3 ligases attach ubiquitin chains to target proteins for subsequent degradation 130. Histone modifications that impact E3 expression can modify the post-endocytosis fate of DAT. The expression of deubiquitinating enzymes is also regulated by histone modifications, facilitating the removal of ubiquitin chains and enabling DAT recycling rather than degradation. Acetylation of CBP/p300 activates genes associated with E3 ubiquitin ligase (such as NEDD4-2), which enhances DAT ubiquitination, marking it for endocytosis and subsequent degradation 131. By inhibiting the acetylation of these enzymes, it is possible to decrease ubiquitination and enhance DAT recycling. Histone modifications also play a role in regulating the efficiency of DAT endocytosis and recycling by controlling the synthesis of proteins associated with cytoskeleton dynamics, membrane transport, and endocytic machinery. These proteins are essential for the formation of endosomes, transportation of endocytic vesicles, and vesicle recycling. Histone modifications regulate the expression of genes involved in cytoskeletal dynamics, such as small GTPase proteins and microtubule-associated proteins, thereby modulating DAT endocytosis through the regulation of endosome formation and transport. Small molecules that inhibit histone deacetylation activate the expression of endocytic proteins like Dynamin and Rab GTPase, enhancing cellular endocytic mechanisms and promoting DAT endocytosis and recycling. Inhibition of KDM5B can remove H3K4 methylation, leading to the activation of genes related to Dynamin and Clathrin, thus facilitating the formation and transport of endocytic vesicles and promoting DAT endocytosis. Histone modifications also impact the activation state of nuclear receptors or transcription factors, thereby indirectly regulating the expression of proteins involved in the process of DAT endocytosis. Histone acetyltransferases (such as CBP/p300) can activate the expression of genes related to endocytosis by acetylating specific nuclear receptors (e.g., NFκB) 132. The binding of nuclear receptors to target genes promotes the synthesis of proteins involved in endocytosis and phagocytosis processes, thereby regulating DAT endocytosis and recycling. Histone modifications also exert an influence on the activity of transcription factors (such as CREB), which can directly regulate DAT gene expression and the mechanisms underlying membrane protein endocytosis. Acetylation of CREB by CBP/p300 can activate genes associated with membrane protein endocytosis, thus facilitating DAT endocytosis and potentially enhancing its recycling in the postsynaptic region.

Indirect pathways, such as chromatin remodeling, signal transduction cascades, ubiquitination and deubiquitylation processes, regulation of cytoskeleton dynamics, as well as modulation of nuclear receptors and transcription factors, exert influence on the endocytosis of the DAT. These intricate pathways intricately regulate the expression and activity of proteins involved in endocytosis, thereby indirectly determining the processes of endocytosis, recycling, and degradation of DAT. Consequently, these mechanisms provide additional layers of regulation for optimizing DAT functionality on neuronal membranes.

### The impact of small molecule inhibitors on histone signaling alterations influences the process of DAT endocytosis

Small molecule inhibitors regulate DAT endocytosis by modulating histone modifications. These inhibitors exert their effects on the expression of genes and proteins involved in endocytosis, thereby influencing the localization, rate of internalization, and recycling patterns of DAT on the cellular membrane. The following elucidates how small molecule inhibitors control DAT endocytosis through histone signaling mechanisms. HDAC inhibitors, one of the extensively investigated small molecule inhibitors, impedes histone deacetylation process to elevate acetylation levels that facilitate chromatin relaxation and enhance gene transcription. These inhibitors enhance the expression of genes associated with DAT endocytosis and membrane transport by elevating acetylation levels. The process of DAT endocytosis relies on various proteins involved in endocytosis, such as Clathrin, the AP-2 complex, and Dynamin. HDAC inhibitors promote the synthesis of these proteins, thereby facilitating DAT endocytosis. Trichostatin A (TSA), a commonly used HDAC inhibitor, prevents histone H3 and H4 deacetylation, leading to increased acetylation levels. By inducing chromatin structure relaxation, TSA activates the expression of genes related to endocytosis, thus promoting DAT endocytosis and recycling 133. The HDAC inhibitor Vorinostat (such as SAHA) can also enhance the expression of endocytosis-regulating proteins, such as Dynamin and Clathrin, thereby facilitating the internalization of DAT from the cellular membrane 134. HDAC inhibitors facilitate chromatin relaxation and opening by modulating histone modification states, thereby inducing the activation of endocytosis-related genes, enhancing DAT endocytosis rate, and potentially expediting post-synaptic DAT recycling. Conversely, HAT inhibitors suppress histone acetyltransferase activity, leading to reduced levels of acetylation and resulting in more compacted chromatin structure and repression of gene expression. HAT inhibitors can attenuate the expression of genes involved in the process of DAT endocytosis, decelerating endocytosis. By inhibiting the acetylation of histones, HAT inhibitors decrease the expression of genes related to endocytosis, resulting in a deceleration of the rate at which endocytosis occurs. They impact the synthesis of crucial proteins associated with endocytosis, phagocytosis, and vesicle formation, thereby reducing DAT endocytosis in neuronal membranes. C646, a CBP/p300 HAT inhibitor, restricts chromatin accessibility by suppressing histone acetylation and subsequently downregulating the expression of proteins involved in regulating endocytosis such as Clathrin, Dynamin, and Rab GTPase 135. By downregulating the expression of these proteins, C646 can attenuate the rate of DAT endocytosis and recycling. HAT inhibitors diminish chromatin accessibility by inhibiting acetylation, thereby reducing the expression of genes involved in the endocytosis process. This decelerates DAT endocytosis and potentially prolongs its residence time on the presynaptic membrane. HMT inhibitors modulate gene expression and chromatin structure through inhibition of histone methylation. These inhibitors indirectly impact the endocytosis process by regulating the expression of genes associated with DAT endocytosis. The inhibition of HMT enzymes impedes histone methylation, particularly inhibitory marks such as H3K27Me3, thereby alleviating the repression on genes related to endocytosis. This leads to an upregulation in the expression of proteins involved in regulating endocytosis and subsequently impacts DAT endocytosis. GSK126, an inhibitor targeting EZH2, disrupts the formation of H3K27Me3, thus relieving the repression on genes associated with both endocytosis and phagocytosis and promoting their transcription. The expression of these genes may enhance the rate of DAT endocytosis and impact its dynamic regulation on the presynaptic membrane. DZNep, an additional inhibitor of EZH2 136. The reduction of H3K27Me3 marks enhances the expression of genes related to endocytosis, thereby facilitating DAT endocytosis and recycling 137. HMT inhibitors enhance the endocytosis and recycling of DAT by removing inhibitory methylation marks, such as H3K27Me3, thereby alleviating the repression on genes related to DAT endocytosis and promoting the synthesis of endocytosis proteins. The activation of genes associated with DAT endocytosis can be effectively blocked by these inhibitors, thereby exerting an influence on the rate of DAT endocytosis. KDM inhibitors maintain the methylation status of histones to reduce the activation of genes related to endocytosis 138. By maintaining inhibitory methylation on histones (such as H3K9Me3 and H3K27 Me3), these inhibitors can decrease the production of key proteins involved in the process of endocytosis, thereby reducing the efficiency of DAT endocytosis. GSK-J1 is a KDM6 inhibitor that prevents demethylation, thus preserving the inhibitory methylation status of H3K27Me3 and subsequently decreasing the activation of genes related to endocytosis, ultimately inhibiting DAT endocytosis. As a broad-spectrum inhibitor of KDMs, it impedes the demethylation process of H3K4 and H3K9, leading to a more condensed chromatin state and downregulation of genes associated with endocytosis. Consequently, this diminishes the rate at which DAT is internalized 139. The inhibition of KDM enzymes maintains methylation marks that reduce chromatin accessibility and downregulate the expression of genes associated with DAT endocytosis, thereby potentially impeding the processes of DAT endocytosis and recycling.

HDAC and HAT inhibitors enhance DAT endocytosis by regulating gene expression. HMT inhibitors promote DAT endocytosis by reducing inhibitory methylation marks and facilitating the expression of endocytosis-related genes. KDM inhibitors suppress DAT endocytosis by maintaining inhibitory methylation levels and attenuating the activation of endocytosis-related genes. The inhibitors serve as crucial pharmacological instruments for manipulating the functions of neurotransmitter transporters.

## The endocytosis of the DAT is influenced by chromatin remodeling

Chromatin remodeling exerts a regulatory influence on the expression of numerous genes, governing neuronal survival and death, and plays a pivotal role in the onset and development of PD. The process of chromatin remodeling primarily depends on the activity of chromatin remodeling complexes, which utilize energy obtained from ATP hydrolysis to modify the position or conformation of nucleosomes, thereby regulating the accessibility of DNA. These complexes are capable of sliding nucleosomes to expose or conceal specific regions of DNA, thus facilitating or impeding the access of transcription factors to DNA sequences and subsequently influencing gene expression. The function of certain chromatin remodeling complexes involves the complete removal of nucleosomes from DNA, thereby fully exposing DNA sequences for transcription. Through the replacement or rearrangement of histone subunits, these complexes can alter the properties of nucleosomes and subsequently impact gene regulation. Additionally, remodeling complexes possess the ability to modulate the strength of DNA-histone binding, thus regulating the open or closed state of chromatin. Chromatin remodeling complexes are multiprotein assemblies, typically composed of a catalytic subunit and multiple regulatory subunits. The SWI/SNF family represents one of the earliest identified families of chromatin remodeling complexes, capable of modulating gene expression through nucleosome sliding or eviction. The SWI/SNF complex is closely associated with tumorigenesis, as mutations in its constituents have been identified in various types of cancer. The ISWI family of remodeling complexes primarily regulates chromatin structure through nucleosome sliding and plays a crucial role in chromatin assembly and transcriptional regulation 140, 141. The Chromodomain Helicase DNA-binding (CHD) family possesses chromatin remodeling functions and encompasses chromodomain motifs capable of recognizing histone modifications, thereby exerting regulatory control over gene expression accordingly 142. The INO80/SWR1 family regulates processes such as DNA damage repair and replication through the reorganization of nucleosomes or the replacement of histone variants.

The conformation of chromatin can be modified by chromatin remodeling complexes, which enables precise control of gene activation or silencing. During gene activation, these complexes facilitate the relaxation of chromatin structure, thereby granting access to transcription factors and RNA polymerase for initiating transcription 143. Conversely, during gene silencing, chromatin remodeling complexes condense the chromatin structure, rendering it challenging for transcription factors to access the DNA and thus suppressing gene expression. Aberrations in chromatin remodeling are frequently associated with various diseases, particularly cancer and neurodegenerative disorders. Mutations or dysfunctions in chromatin remodeling complexes can contribute to the development of cancer. Mutations in the SWI/SNF complex have been identified in multiple tumors, potentially facilitating cancer progression by altering the expression of tumor suppressor genes or oncogenes 144. The involvement of specific chromatin remodeling complexes in the pathological processes of these diseases, through their regulation of genes associated with neuronal survival and function, is increasingly recognized. Given the pivotal role played by chromatin remodeling in disease pathogenesis, therapeutic strategies targeting these complexes are emerging as promising interventions 105. The primary mechanism of action for HDAC inhibitors can also indirectly influence chromatin remodeling, leading to advancements in their application for specific cancer treatments 145. Some small molecule inhibitors are currently being developed to target specific subunits of chromatin remodeling complexes, with the aim of intervening in the progression of cancer and other diseases. Chromatin remodeling plays a pivotal role in regulating gene expression by modifying chromatin structure, thereby enabling cells to respond effectively to diverse environmental stimuli. The primary role of chromatin remodeling lies in its regulation of the expression of key genes in neurons, thereby exerting a significant influence on various pathological processes associated with PD, including neuroinflammation, apoptosis, and mitochondrial dysfunction 146. The expression of disease-related genes is influenced by chromatin remodeling complexes, which regulate the accessibility of chromatin 147. The involvement of these complexes in inflammatory responses is mediated through the modulation of neuroinflammation-related gene expression. Certain remodeling complexes may aggravate neuronal damage by inducing the expression of inflammatory factors via alterations in chromatin structure. Additionally, certain chromatin remodeling complexes have the ability to regulate the expression of neuroprotective genes, including anti-apoptotic and antioxidant genes, which are frequently suppressed in PD and may be associated with aberrant chromatin remodeling 148. The malfunction of these complexes, which serve as the executors of the chromatin remodeling process, may contribute to the initiation or exacerbation of PD.

### Relevant enzymes influencing the endocytosis of DAT during chromatin remodeling

The ATP-dependent chromatin remodeling complexes, such as SWI/SNF and NuRD complexes, utilize energy to modulate nucleosome positioning, thereby regulating gene accessibility and expression 149. The presence of these complexes can indirectly impact the expression of genes associated with DAT endocytosis. Chromatin remodeling complexes play a regulatory role in repositioning nucleosomes, thereby controlling chromatin accessibility and ultimately influencing the transcriptional activity of genes involved in endocytosis and internalization. The expression of these genes will directly impact the efficiency of DAT endocytosis and recycling. BRG1/BRM, as core ATPases of the SWI/SNF complex, can activate genes associated with endocytosis regulation by remodeling chromatin, thereby promoting DAT endocytosis 150. The presence of CHD4 within the NuRD complex leads to a reduction in transcription activity of genes related to endocytosis by inhibiting chromatin accessibility, thereby effectively suppressing DAT endocytosis 151. The SWI/SNF complex enhances DAT endocytosis through gene activation, while the NuRD complex attenuates endocytosis by repressing gene expression. By modulating the accessibility of chromatin, they directly influence the expression of genes related to endocytosis. Through their regulation, these complexes indirectly control the rate of DAT endocytosis and recycling patterns, providing a crucial epigenetic checkpoint for neurotransmitter transporter regulation.

### The DAT endocytosis process is regulated by indirect pathways involved in chromatin remodeling

These indirect routes often involve a series of signaling cascades, transcription factors, and regulatory proteins that ultimately modulate this process by impacting the expression or activity of genes related to DAT endocytosis. The regulation of chromatin remodeling is governed by multiple cellular signaling pathways, including the cAMP-PKA, MAPK, and PI3K-Akt pathways. These signals can indirectly impact DAT endocytosis by modulating the activity of chromatin remodeling complexes and epigenetic enzymes. For instance, the cAMP-PKA pathway phosphorylates various transcription factors and subunits of chromatin remodeling complexes to regulate the accessibility of chromatin 152. This pathway can exert an influence on the transcriptional activity of genes associated with endocytosis and internalization, thereby indirectly regulating DAT endocytosis. Following PKA-mediated phosphorylation of CREB, the activated CREB interacts with chromatin remodeling complexes to facilitate the transcription of pertinent genes, augmenting the expression of pivotal proteins essential for DAT endocytosis. The MAPK pathway can also modulate the expression of genes related to endocytosis by activating transcription factors such as Elk-1 and AP-1, thereby modifying their interactions with chromatin remodeling complexes 153. This regulation can indirectly impact the process of DAT endocytosis. Under the influence of the ERK pathway, Elk-1 activates transcription of specific genes, including proteins associated with endocytosis such as clathrin and Dynamin, thereby facilitating DAT endocytosis. The PI3K-Akt pathway indirectly modulates chromatin remodeling by regulating transcription factors like FOXO, which control the expression of genes related to endocytosis 154. After Akt is activated, it phosphorylates and inhibits FOXO transcription factors, reducing their repressive effect on chromatin remodeling complexes, thereby indirectly promoting the expression of genes related to DAT endocytosis. Some key transcription factors directly bind to chromatin remodeling complexes, regulating the expression of endocytosis-related genes and influencing DAT endocytosis. The activity of these transcription factors can be modulated by various intracellular signals. Once phosphorylated, CREB (cAMP response element-binding protein) interacts with histone acetyltransferases (such as CBP/p300), facilitating chromatin relaxation and activating the expression of genes associated with DAT endocytosis. The interaction between CREB and CBP enhances histone acetylation, thereby facilitating the activation of gene expression, such as clathrin and Dynamin, which subsequently reinforces the process of DAT endocytosis 155. The transcription factor NFκB plays a crucial role in mediating inflammatory responses and exhibits the ability to interact with chromatin remodeling complexes, thereby regulating the expression of genes associated with membrane endocytosis. In response to extracellular stress signals, NFκB can activate specific endocytic genes, thereby enhancing DAT endocytosis. The FOXO family of transcription factors directly bind to chromatin remodeling complexes, resulting in the inhibition of certain endocytic genes. Their activity is negatively regulated by the PI3K-Akt pathway. Phosphorylation of FOXO by At and its subsequent transport out of the nucleus relieves its inhibitory effect on endocytic genes, thereby promoting DAT endocytosis. Chromatin remodeling complexes, such as SWI/SNF and NuRD, regulate the expression of genes involved in DAT endocytosis. The SWI/SNF complex modulates nucleosome positioning to modify gene accessibility, indirectly impacting chromatin structure remodeling and enhancing the accessibility of specific endocytosis-regulating genes, thereby promoting DAT endocytosis. BRG1, a core subunit of the SWI/SNF complex, is capable of activating genes involved in endocytosis, thereby enhancing DAT internalization. Conversely, the NuRD complex exerts inhibitory effects on gene expression through deacetylation and chromatin compaction processes, leading to reduced expression of endocytic genes and consequent inhibition of DAT endocytosis. The CHD4 protein, a critical component of the NuRD complex, functions to repress chromatin accessibility, thereby reducing the synthesis of proteins essential for endocytosis and subsequently decreasing DAT endocytosis rates.

Chromatin remodeling regulates DAT endocytosis through various indirect pathways, including signal transduction cascades (such as cAMP-PKA, MAPK, and PI3K-Akt), transcriptional regulators (such as CREB, NF-κB, and FOXO), and epigenetic modifications (such as histone acetylation or methylation). Chromatin remodeling complexes, such as SWI/SNF and NuRD, along with non-coding RNAs like miRNA and lncRNA, are also intricately involved in influencing the expression of genes associated with DAT endocytosis. Through these intricate regulatory networks, chromatin remodeling processes can precisely modulate the dynamics of DAT internalization (Table 2).

### The involvement of small molecule inhibitors in the process of DAT endocytosis during chromatin remodeling

The expression of genes or the activity of proteins associated with DAT endocytosis can be indirectly regulated by small molecule inhibitors through targeting chromatin remodeling complexes, epigenetic modification enzymes, or key proteins in signaling pathways. These inhibitors can modulate chromatin accessibility, thereby impacting DAT endocytosis. Several key classes of small molecule inhibitors can target the core proteins of chromatin remodeling complexes (such as SWI/SNF, NuRD, etc.), which utilize ATP to regulate gene expression by changing chromatin structure. Modulating chromatin openness through these inhibitors indirectly affects genes related to DAT endocytosis. The function of chromatin remodeling complexes is hindered by inhibitors that specifically target core subunits (such as BRG1, BRM, etc.), thereby obstructing the process of chromatin remodeling and indirectly impacting the expression of genes associated with DAT endocytosis. PFI-3, a selective inhibitor of SMARCA2/4 (BRG1/BRM), effectively blocks the activity of the SWI/SNF complex, resulting in reduced expression levels of genes related to endocytosis and consequently decreasing DAT endocytosis. Inhibitors targeting chromatin remodeling complexes can attenuate the transcription of genes associated with DAT endocytosis by impeding the functionality of these complexes, thereby indirectly inhibiting the process of endocytosis. The PI3K-Akt pathway assumes a pivotal regulatory role in chromatin remodeling. By suppressing this pathway, small molecule inhibitors can indirectly modulate the state of chromatin remodeling and regulate DAT endocytosis. The activation of PI3K or Akt is blocked by inhibitors targeting the PI3K-Akt pathway, resulting in reduced phosphorylation of downstream transcription factors such as FOXO. This leads to their accumulation in the nucleus and subsequent inhibition of endocytosis-related gene transcription. LY294002, a well-established PI3K inhibitor, effectively suppresses PI3K activity and attenuates Akt activation, thereby indirectly inhibiting the expression of genes associated with DAT endocytosis 156. The PI3K-Akt pathway inhibitors impede signaling, thereby diminishing the expression of genes associated with DAT endocytosis and hindering the process of endocytosis.

The reprogramming of pluripotent stem cells (iPSCs) and the differentiation, apoptosis, and death of DAergic neurons can be modulated by small molecule inhibitors and natural bioactive compounds through the regulation of epigenetic processes. These substances exhibit promising therapeutic effects on PD and offer innovative treatment strategies. The literature has documented that specific small molecule inhibitors or natural bioactive compounds have the potential to counteract the reprogramming of induced iPSCs and aberrant differentiation, apoptosis, and demise of DAergic neurons by modulating epigenetic processes. Consequently, these findings hold promise for the treatment of PD 157.

**Figure 3:**
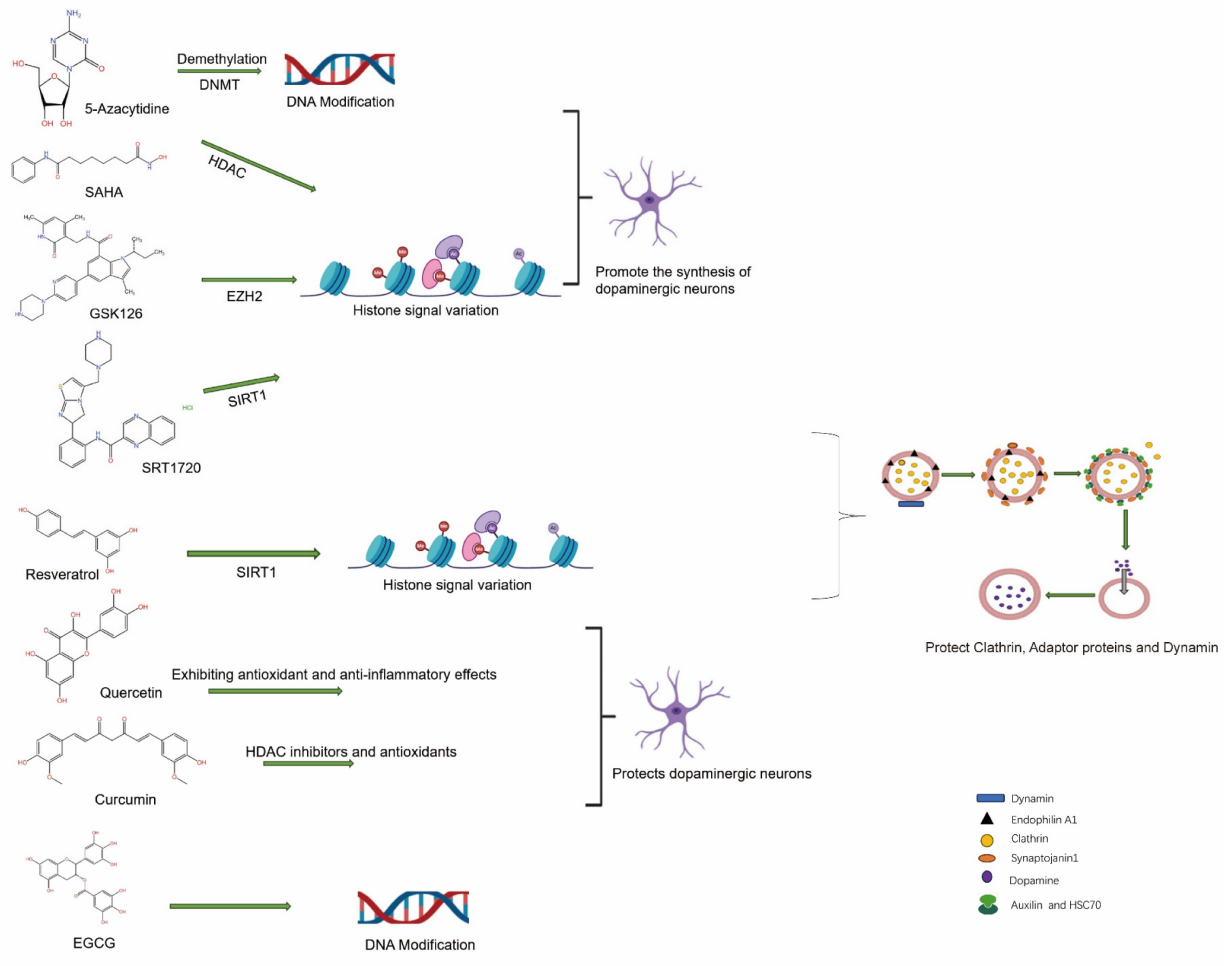
** The impact of epigenetic modifications on the endocytosis of DAT is directly or indirectly influenced by small molecule inhibitors and natural active substances.** SAHA can increase histone acetylation levels by inhibiting HDAC; EZH2 inhibitors prevent the formation of H3K27Me3; SIRT1 activators increase histone deacetylation levels; SIRT1 activators increase histone deacetylation levels; EGCG DNA methyltransferase inhibitors and antioxidants.

By modulating DAergic neurons through epigenetic pathways, small molecule inhibitors and naturally occurring active substances may provide novel therapeutic options for PD. The compounds possess the ability to safeguard existing DAergic neurons, decelerate their degeneration process, facilitate nerve regeneration or repair mechanisms, and regulate genes associated with inflammatory responses or oxidative stress. Consequently, these actions contribute to the alleviation of symptoms related to PD (Figure 3, Table 3).

Particularly in relation to PD-associated DAT endocytosis, epigenetic regulation plays a critical role 158. Epigenetic modifications can directly influence the expression of the DAT gene itself, such as through DNA methylation and histone modifications. The regulation or aberrant activation of the DAT gene impacts dopamine uptake, storage, and release, thereby affecting it signaling. Epigenetic factors can indirectly modulate the endocytic efficiency of DAT by regulating the expression and activity of key enzymes involved in endocytosis. Alterations in endocytic efficiency can impact the rate of dopamine reuptake, subsequently influencing its levels within the synaptic cleft. Dysregulation of DAT endocytosis and degradation may result in abnormal accumulation or depletion of DA within the synaptic cleft. The dysregulation of DA signaling is widely acknowledged as a pivotal factor in the initiation and progression of PD. Consequently, the involvement of epigenetic regulation in PD may exert either facilitative or inhibitory effects on these crucial processes, thereby modulating disease progression.

## Conclusion and Prospects

Epigenetic mechanisms such as DNA methylation, histone modifications, and regulation of non-coding RNA can impact DAT endocytosis by influencing neuronal survival, thereby contributing to the development of PD. Small molecule inhibitors or bioactive compounds have the potential to modulate these processes, thereby reversing iPSC reprogramming and abnormal differentiation, offering promising therapeutic strategies. Although there is no specific DNA modification enzyme directly controlling DAT endocytosis, DNMTs and m^6^A methyltransferases may indirectly influence it through the regulation of gene expression. The modifications exert an impact on crucial signaling pathways such as PKC and D2R, thereby influencing the processes of protein synthesis and chromatin accessibility. Consequently, this indirect regulation plays a pivotal role in controlling DAT endocytosis and recycling. Targeting these intricate mechanisms holds promising therapeutic potential for early-stage PD. Additionally, there are certain aspects that necessitate further discussion.

Notably, epigenetic modifications play a significant role in the pathogenesis of PD by precisely regulating gene expression. Specifically, these modifications can impact on the activity of crucial enzymes involved in the endocytic pathway of the DAT, thereby modulating the pathological progression of PD by regulating DAergic neuron differentiation and function. The key enzymes, including protein kinases and phosphatases involved in the endocytic pathway, play a crucial role in regulating the efficiency of DAT endocytosis and recycling. Aberrant epigenetic modifications can disrupt the function of these enzymes, leading to an imbalance in DA levels within DAergic neurons and increasing neurotoxicity. Furthermore, epigenetic modifications can exert an influence on the expression of genes associated with neuronal differentiation, thereby impacting the development and functionality of DAergic neurons. Aberrant epigenetic alterations may impede or redirect neuronal differentiation, leading to impaired normal neuronal function and decreased survival rate of DAergic neurons, consequently facilitating the progression of PD. Epigenetic modifications may exacerbate the pathological features of PD, such as the loss of DAergic neurons and the abnormal accumulation of SNCA, by influencing DAT endocytosis and neuronal differentiation. Given the significance of epigenetic modifications in PD, therapeutic strategies targeting these modifications (such as epigenetic drugs) have potential to restore normal DAT endocytosis and neuronal differentiation functions, thereby decelerating disease progression. The later disease primarily affects the regions of the brain responsible for motor control 159. Aberrant methylation patterns of multiple disease-associated genes have been detected in the brain tissue of PD individuals. For instance, the atypical expression of the SNCA gene is closely linked to the pathogenesis of PD 160. SNCA is primarily localized at the presynaptic terminals of neurons, where it is situated in the cytoplasm and partially associated with synaptic vesicles and the cell membrane. It plays a crucial role in regulating synaptic vesicle recycling, neurotransmitter release, as well as maintaining membrane stability and plasticity. Additionally, it interacts with membrane phospholipids, vesicles, and cytoskeletal proteins to effectively modulate synaptic activity. The abnormal folding of SNCA leads to the formation of insoluble fibrils, which are the primary constituents of Lewy bodies and Lewy neurites. These aggregates can be observed in various cellular compartments including cell bodies, axons, and dendrites. Their presence and distribution serve as reliable markers for DAergic neuron death, representing a pathological hallmark of PD. The aggregated form of SNCA exhibits neurotoxicity and can be actively secreted into the extracellular matrix, facilitating intercellular propagation and inducing SNCA aggregation in neighboring neurons. Consequently, this cascade triggers mitochondrial dysfunction, endoplasmic reticulum stress, disruption of calcium homeostasis, impairment of autophagy and lysosomal systems, as well as synaptic dysfunction. The combined impact of these effects accelerates neurodegenerative processes, ultimately leading to neuronal demise and exacerbating the progression of the disease^63^. SNCA can indirectly modulate the expression of the DAT gene through interactions with transcription factors or signaling pathways; however, the precise underlying mechanisms remain incompletely elucidated. Research suggests that overexpression or aggregation of SNCA may lead to a downregulation of DAT expression. Additionally, it has been observed that SNCA directly interacts with DAT, thereby influencing its function at the presynaptic membrane. Furthermore, SNCA may enhance the functional activity of DAT and facilitate DA reuptake. However, the aberrant aggregation of SNCA can disrupt normal DAT function, leading to a reduction in the efficiency of DA reuptake and impacting the maturation and transport of endocytic vesicles. As a result, there is dysregulation in DA metabolism, potentially causing an accumulation or depletion of DA in the synaptic cleft that ultimately affects neurotransmission. The involvement of SNCA in the internalization of DAT and the formation of endocytic vesicles is postulated. Abnormal aggregation of SNCA and its interference with DAT function and endocytic processes are considered pivotal mechanisms underlying neuronal death in PD. Dysfunction of DAT can induce aberrant alterations in DA levels within the synaptic cleft, thereby triggering oxidative stress, inflammatory responses, and subsequent neurodegenerative changes. The extracellular propagation of SNCA aggregates may lead to the pathological spread to additional neurons, progressively affecting larger regions of the brain. This propagation further exacerbates the onset and progression of PD 161. The role of SNCA encompasses not only its aberrant aggregation into toxic fibrils but also its interaction with the DAT and regulation of endocytic processes, thereby impacting the dynamic equilibrium of DA. These alterations play a pivotal role in the pathological progression of PD. The research suggests that a decrease in methylation levels within the promoter region of the SNCA gene results in an upregulation of expression, consequently promoting neuronal damage and death 162. The DJ-1 gene plays a neuroprotective role in PD, and alterations in methylation within its promoter region may impact its functionality, potentially hastening the progression of the disease 163.

m⁶A may exert regulatory control over the protein levels of alpha-synuclein by modulating its gene expression, thereby contributing to the pathogenesis of PD. Neuroinflammation is a significant contributor to neuronal loss, and m⁶A modifications have the potential to modulate inflammatory responses by regulating the expression of inflammation-related genes. This includes exerting influence on the stability and translation efficiency of SNCA mRNA, which in turn governs SNCA expression and plays a pivotal role in PD pathology. The mRNA expression of markers such as TH and FOXA2 can be regulated by m⁶A, thereby influencing neuronal differentiation 164. Apart from m⁶A, other forms of RNA modifications may also be implicated in PD. Pseudouridine modifications serve to stabilize tRNA and rRNA, while also regulating protein synthesis. Disruptions in these modifications within neurons may have an impact on protein synthesis and function, ultimately affecting neuronal survival and functionality. The role of m⁵C in RNA is still being explored; however, studies suggest that it plays a significant role in the regulation of gene expression and RNA processing. The alterations in m⁵C may exert an impact on the expression of genes associated with diseases 165. RNA modification enzymes play a crucial role in adding chemical groups to RNA molecules. Abnormal expressions or dysfunctions of these enzymes in PD can disrupt the regulation of relevant RNA modifications, subsequently impacting neuronal function. The METTL3/METTL14 complex is involved in modifying and regulating key genes associated with PD, potentially influencing neuronal survival and inflammatory responses 166. Reversible dysregulation of m⁶A demethylases, such as FTO and ALKBH5, may result in aberrant gene expression associated with PD 167. Targeting regulators of RNA modifications may present a potential therapeutic strategy, such as modulating m⁶A levels or adjusting the activity of associated modification enzymes, which could potentially attenuate neuronal loss and ameliorate symptoms.

Alterations in histone signaling play a pivotal role in the pathological mechanisms of PD. Changes in histone modifications may contribute to the initiation and progression of this disorder by exerting regulatory control over gene expression and chromatin architecture. The acetylation of histones is commonly associated with the activation of gene expression. In neurons of patients with PD, aberrant gene expressions may be linked to dysregulation in histone acetylation. It is possible that the activity of HDACs could be elevated in PD 168. The suppressed expression of neuroprotective genes, such as antioxidant and anti-apoptotic genes, can result in neuronal damage and death. Experimental models have demonstrated that HDAC inhibitors exhibit certain neuroprotective effects. By inhibiting HDAC activity, it is possible to restore histone acetylation levels and enhance the expression of neuroprotective genes, thereby attenuating neuronal loss. The role of histone methylation in regulating gene expression is multifaceted, as it can either activate or repress gene expression depending on the specific modification sites. Studies have revealed altered levels of certain H3 methylation sites (such as H3K4Me3 and H3K27Me3) in the brains of patients with PD 169. These changes may impact the expression of genes associated with neuroinflammation, apoptosis, and neuronal survival. Altered activity of specific histone demethylases, such as LSD1 and JMJD3, has also been implicated in the pathology of PD 170, 171. They may exert an impact on neuronal function by modulating the methylation status of specific genes. Histone phosphorylation is linked to DNA damage repair and cell cycle regulation. Oxidative stress and DNA damage play pivotal roles in neuronal demise. In PD models, levels of phosphorylated histone H2A.X (γ-H2A.X) are elevated, indicating the presence of DNA damage in neurons 172. The phosphorylation modification can recruit DNA repair proteins. However, persistent oxidative stress may overwhelm the DNA repair mechanisms, resulting in further neuronal damage. SNCA has the potential to modulate histone modification states through interactions with histone modifying enzymes, subsequently impacting gene expression. The interaction between SNCA and HDACs may exert an influence on the acetylation status in neurons, thereby facilitating disease progression. Moreover, SNCA might modulate the activity of HMTs, leading to alterations in the methylation status of crucial genes and consequently impacting neuronal fate.

The histone modifications H3K27Me3 and H3K27Ac are distinct and respectively associated with gene silencing and activation processes 173. The roles of these factors in epigenetics typically exhibit opposing effects, but their influence on specific biological processes may be relatively complex. H3K27Me3 is usually mediated by PRC2 and often serves as an epigenetic mark for gene silencing or transcriptional repression. The elevation of H3K27Me3 levels may impede the transcription of genes associated with DAT expression or endocytic regulation, thereby exerting an influence on DAT levels or activity. Consequently, this indirect effect could lead to a reduction in DAT expression or its presence on the cell membrane, resulting in a decrease in endocytic demand. Conversely, H3K27Ac is predominantly associated with enhancer regions and actively transcribed genes, indicating their ongoing transcriptional activity. An increase in H3K27Ac levels may facilitate the expression of DAT-related genes, thereby enhancing DAT's localization on the cell membrane and subsequently influencing its endocytosis rate. The frequency of endocytosis of active DAT may be higher. H3K27Me3 and H3K27Ac are inherently mutually exclusive modifications and cannot coexist at the same site. H3K27Me3 marks gene silencing, while H3K27Ac indicates gene activity 174. Thus, their effects on the same gene or genomic region typically exhibit opposite regulatory outcomes. Disruption of the delicate balance between these modifications on genes or regulatory factors governing DAT endocytosis could result in distinct regulatory states of endocytosis. Specifically, elevated levels of H3K27Me3 may potentially suppress the expression of endocytosis-related genes, while increased levels of H3K27Ac might promote their expression 175. The contradictions may arise from several factors: Firstly, the specificity of cell types. H3K27Me3 and H3K27Ac may exert distinct regulatory effects depending on the specific cellular environment, other regulatory factors, and signaling pathways in neurons or DAergic cells. Additionally, temporal fluctuations in modification levels may exert distinct effects on various stages of DAT endocytosis. The activation of relevant genes might be facilitated by H3K27Ac during the preparation for endocytosis, whereas gene silencing to restore homeostasis could involve H3K27Me3 after the completion of endocytosis 176. The regulation of DAT endocytosis involves not only epigenetic modifications but also multiple signaling pathways, such as the cAMP-PKA and ERK pathways. The indirect regulation of DAT endocytosis by H3K27Me3 and H3K27Ac through distinct signaling pathways may result in contradictory effects. In the presence of specific experimental data or conflicting evidence in the literature, a more comprehensive analysis can be conducted to explore the intricate relationship between epigenetic modifications and DAT endocytosis. The significant role of histone modifications in PD suggests that drugs targeting histone modifying enzymes hold potential therapeutic prospects. By inhibiting HDACs, it may be feasible to restore the expression of neuroprotective genes and provide neuroprotection. The utilization of inhibitors that specifically target methylation states could potentially facilitate the restoration of normal gene expression patterns and decelerate disease progression.

Dysfunction of the SWI/SNF complex may impede the expression of genes associated with neuronal survival, thereby expediting neuronal apoptosis. The ISWI complex plays a pivotal role in nucleosome sliding and chromatin assembly 177. The dysfunction of this process may impact DNA repair and gene expression, thereby further compromising neuronal function. Chromatin remodeling complexes belonging to the CHD family possess chromodomain regions that can recognize modifications in histones and regulate the expression of genes. CHD complexes may modulate disease progression by regulating the expression of pivotal neuronal genes 178. The aberrant aggregation of SNCA represents one of the pathological hallmarks observed in PD. This anomalous accumulation of SNCA may directly or indirectly impact on the functionality of chromatin remodeling complexes, thereby leading to abnormal alterations in chromatin structure. The alterations may further lead to the disruption of neuronal gene expression, thereby facilitating neuronal damage. The aggregation of SNCA has the potential to enhance chromatin compaction, consequently suppressing the expression of specific neuroprotective genes and exacerbating neurodegeneration 179.

Environmental factors, such as pesticides and heavy metals, are recognized as risk factors for PD. These environmental agents have the potential to modulate gene expression by impacting chromatin remodeling processes. Specifically, certain toxins in the environment can induce chromatin compaction, thereby suppressing the expression of neuroprotective genes and increasing neuronal susceptibility. Oxidative stress is a prevalent pathological characteristic that can potentially modulate the activity of chromatin remodeling complexes, thereby inducing alterations in chromatin states and subsequently impacting gene expression and cellular fate. Given the pivotal role played by chromatin remodeling, targeting these complexes with pharmacological agents holds promising therapeutic implications. The utilization of specific small-molecule inhibitors to target chromatin remodeling complexes represents a promising approach for restoring normal gene expression and potentially attenuating the progression of diseases. Epigenetic therapies, such as modulating chromatin remodeling processes using small molecules, present an innovative treatment strategy by enhancing neuronal function.

The focus of future research should be directed towards the development of innovative small molecule drugs that target key enzymes, such as DNMTs, HDACs, and HATs, to effectively regulate epigenetic states and restore the functionality of DAergic neurons. Developing multi-layered regulatory network models is crucial for gaining a comprehensive understanding of the integrated role of epigenetics in PD and investigating the dynamic changes of these mechanisms at different pathological stages. Employing epigenetic markers for patient stratification enables the development of highly personalized therapeutic strategies, optimizing treatment efficacy, attenuating disease progression, and potentially reversing pathological processes. Through a comprehensive examination of these epigenetic mechanisms, we can enhance our comprehension of the pathological processes associated with PD, provide theoretical substantiation for the development of innovative therapeutic approaches, and potentially achieve efficacious intervention and management of neurodegenerative disorders in the future.

## Figures and Tables

**Table 1:** Key enzymes implicated in the endocytic process of DAT and their respective functions.

Name	Type	Main Function	Action Steps
SYNJ1 (Synaptojanin 1)	Phosphatidylinositol phosphatase	Dephosphorylates PI (4,5) P_2_ to PI (4) P, promoting the disassembly of the clathrin coat	Vesicle uncoating
Auxilin	Cofactor	Binds to Hsc70 and recruits it to the clathrin coat, driving ATP hydrolysis to depolymerize clathrin	Vesicle uncoating
PI (4,5) P_2_ (Phosphatidylinositol 4,5-Bisphosphate)	Phospholipid	Recruits clathrin and adapter proteins to promote clathrin coat assembly and regulate dynamin activity 180	Vesicle formation and scission
Dynamin	GTPase	Drives the constriction and scission of the neck of endocytic vesicles through GTP hydrolysis	Vesicle scission
AP-2 (Adaptor Protein Complex 2)	Adapter protein complex	Binds to PI (4,5) P_2_ and endocytic signaling sequences, linking clathrin to membrane receptors to form clathrin-coated pits	Vesicle formation
Hsc70 (Heat Shock Cognate Protein 70)	Chaperone protein	With the assistance of Auxilin, hydrolyzes ATP to drive the disassembly of the clathrin coat	Vesicle uncoating
Endophilin& Amphiphysin	Protein	Involved in membrane curvature regulation, stabilizes clathrin-coated pits, and interacts with other endocytic regulatory proteins	Vesicle formation and scission
Phosphoinositide 3-Kinases (PI3K)	Kinase	Convert PI (4,5) P_2_ to PI (3,4,5) P_3_, promoting vesicle maturation and fusion with early endosomes	Vesicle maturation and fusion

**Table 2:** The research focuses on the impact of epigenetic regulation on PD.

Epigenetic regulatory mechanisms	Mechanism	Detailed description	Regulation of key enzymes	Relationship with PD
DNA Modification	^5^mC	^5^mC occurs on the CpG islands in gene promoter regions and is often associated with gene silencing	High levels of methylation may lead to reduced expression of genes such as SYNJ1 and Auxilin, affecting endocytosis efficiency	Excessive methylation of neuroprotective genes may weaken endocytosis, leading to neuronal dysfunction and death
RNA Modification	m^6^A	m^6^A is the most common mRNA modification, regulating mRNA stability, splicing, translation, and degradation	Affect the mRNA stability and translation efficiency of endocytosis-related enzymes, such as SYNJ1 and Dynamin expression	Abnormal m^6^A modification may lead to dysregulation of DAT regulatory protein expression, affecting dopamine signaling and neuronal survival
Histone Signaling Changes	Activation signals (such as H3K27Ac)	H3K27Ac is an active histone modification associated with gene transcription activation	H3K27Ac is present in the promoter regions of genes such as SYNJ1 and Auxilin, enhancing gene expression	A decrease in H3K27Ac levels may reduce the expression of endocytosis-related genes, affecting dopamine transport and neuronal survival
	Inhibition signals (such as H3K27Me3)	H3K27Me3 is a repressive histone modification associated with gene silencing	H3K27Me3 enrichment in the promoter regions of endocytosis-related genes may suppress gene expression	An abnormal increase in H3K27Me3 may lead to the silencing of key genes such as SYNJ1, promoting neurodegenerative changes
Chromatin Remodeling	SWI/SNF Complex	Alter the chromatin structure through ATP-dependent remodeling mechanisms, making specific genes more accessible for transcription	Changing the accessibility of endocytosis-related genes regulates the expression of genes such as SYNJ1 and Auxilin	Dysregulation of chromatin remodeling may lead to reduced endocytic efficiency, affecting neuronal function and survival
	ISWI Complex	Maintain chromatin structure and suppress unnecessary gene expression	Affecting the transcription of endocytosis-related genes may be associated with PD	Inhibition of endocytic mechanisms may exacerbate neurodegenerative changes in neurons
	CHD Family	Regulate gene expression by recognizing specific histone modifications	Affecting genes related to DAT function and the endocytosis process	Abnormal chromatin remodeling may affect dopamine signaling, increasing the risk of neuronal death

**Table 3:** The research results pertaining to small molecule inhibitors and naturally occurring bioactive compounds.

Category	Compound	Mechanism of Action	Research Findings
Small molecule inhibitors	Vorinostat, SAHA	HDAC inhibitors increase histone acetylation levels	Volinostat increases the expression of neuron-specific genes during the differentiation of iPSCs into dopaminergic neurons, improving cell differentiation efficiency and function^134^
	5-Azacytidine	DNA methyltransferase inhibitors reduce DNA methylation levels	5-Aza-cytidine activates the expression of dopaminergic neuron-related genes through demethylation, enhancing the differentiation efficiency of iPSCs into dopaminergic neurons^181^
	GSK126	EZH2 inhibitors prevent the formation of H3K27Me3	GSK126 reduces H3K27Me3 levels by inhibiting EZH2, activating genes related to neuronal differentiation, and improving the differentiation of dopaminergic neurons^182^
	SRT1720	SIRT1 activators increase histone deacetylation levels	SRT1720 improves mitochondrial function and reduces oxidative stress by activating SIRT1, protecting dopaminergic neurons from apoptosis^183^
Natural active substances	Resveratrol	SIRT1 activators increase histone deacetylation levels	Resveratrol protects dopaminergic neurons by activating SIRT1, improving mitochondrial function, and reducing oxidative stress^184^
	Quercetin	Exhibiting antioxidant and anti-inflammatory effects	Quercetin can reduce oxidative stress and inflammation, protecting dopaminergic neurons and improving cell survival and function^185^
	Curcumin	HDAC inhibitors and antioxidants	Curcumin improves the differentiation and survival of dopaminergic neurons by inhibiting HDAC and reducing oxidative stress^186^
	Epigallocatechin Gallate, EGCG	DNA methyltransferase inhibitors and antioxidants	EGCG improves the differentiation and survival of dopaminergic neurons through demethylation and reducing oxidative stress^187^

## Abbreviations

AD: Alzheimer's disease
ADAR: Adenosine Deaminase
BDNF: Brain-Derived Neurotrophic Factor
CDE: Clathrin-Dependent Endocytosis
CHD: Chromodomain Helicase DNA-binding
CIE: Clathrin-Independent Endocytosis
CME: Clathrin-Mediated Endocytosis
DA: Dopamine
DAergic: Dopaminergic
DAT: Dopamine Transporter
DNMTs: DNA Methyltransferases
HATs: Histone Acetyltransferases
HDACs: Histone Deacetylases
HMTs: Histone Methyltransferases
iNOS: Inducible Nitric Oxide Synthase
iPSCs: Induced Pluripotent Stem Cells
KDMs: Histone Lysine Demethylases
MDA: Malondialdehyde
NfL: Neurofilament Light Chain
NSUN2: NOP2/Sun RNA Methyltransferase Family Member 2
PD: Parkinson's Disease
PRC2: Polycomb Repressive Complex 2
PKC: Protein Kinase C
PRMTs: Protein Arginine Methyltransferases
RBPs: RNA-binding proteins
SNCA: α-Synuclein
SYNJ1: Synaptojanin 1
TSA: Trichostatin A
UPS: Ubiquitin-Proteasome System
8-OHdG: 8-Hydroxy-2'-Deoxyguanosine
5-Aza-Dc: 5-Aza-2'-Deoxycytidine
